# Semantic processing of English sentences using statistical computation based on neurophysiological models

**DOI:** 10.3389/fphys.2015.00135

**Published:** 2015-05-22

**Authors:** Marcia T. Mitchell

**Affiliations:** Computer and Information Sciences Department, Saint Peter's UniversityJersey, NJ, USA

**Keywords:** computational linguistics, semantic, iconic neuronal circuits, neuronal network, convergence and divergence zones, attention and consciousness

## Abstract

Computer programs that can accurately interpret natural human language and carry out instructions would improve the lives of people with language processing deficits and greatly benefit society in general. von Neumann in theorized that the human brain utilizes its own unique statistical neuronal computation to decode language and that this produces specific patterns of neuronal activity. This paper extends von Neumann's theory to the processing of partial semantics of declarative sentences. I developed semantic neuronal network models that emulate key features of cortical language processing and accurately compute partial semantics of English sentences. The method of computation implements the MAYA Semantic Technique, a mathematical technique I previously developed to determine partial semantics of sentences within a natural language processing program. Here I further simplified the technique by grouping repeating patterns into fewer categories. Unlike other natural language programs, my approach computes three partial semantics. The results of this research show that the computation of partial semantics of a sentence uses both feedforward and feedback projection which suggest that the partial semantic presented in this research might be a conscious activity within the human brain.

## Introduction

Determining how the human brain processes the meaning of language could be important in helping people with deficits in language comprehension, either because of specific brain disorders dementia or brain lesions (Ullman, 2001; Cooke et al., 2003; Dronkers et al., 2004; Schirmer, 2004; Awad et al., 2007; Sonty et al., 2007; Christensen, 2008; Pulvermüller and Fadiga, 2010; Wright et al., 2012). These insights can inform the development of innovative artificial intelligence that understands and carries out instructions from humans (Pollack, 2005; Russell and Norvig, 2009). Building such intelligent entities has long been the goal of artificial intelligence research (Jurafsky and Martin, 2008).

Current semantic processing models fail to accurately mimic key features of neuronal circuits that process language in the human brain. Currents models fail to accurately show how a sentence would be represented and processed with in a neuronal network.

von Neumann (2000) theorized that the nervous system uses a unique system of notation in which meaning is conveyed via the statistical properties of the message (von Neumann, 2000). I extended this idea to develop a semantic neuronal network model that uses a mathematical language to determine the partial semantics of sentences. The model is informed by both linguistic theory as well as neuroscience research.

In the model each word in a sentence is mapped onto individual neurons whose synchronous activity is summated by a downstream special neuron representing that word category. In this case category refers to word category (i.e., noun or verb). This synchronous activity is modeled after the synchronous behavior of an assembly of neurons in the human brain that represents a given percept or concept (Fell and Axmacher, 2011, p. 105). Thus, the special noun neuron keeps track of the sum of all nouns occurring in the sentence using summation or arithmetic. After all of the summations are collected by these special neurons, they then fire another burst of synchronized spikes. Each special neuron reduces its count by 1 until the verb category is in its lowest term (v^1^).

I simplified the MAYA Semantic Technique to combine repeating word patterns into one of two categories (verbs and noun-phrases). The MAYA Semantic Technique utilizes the semantic neuronal model to determine the verb in a sentence. It also determines the object of the sentence using the same mathematical technique. Categories in a sentence are identified and then used to determine phrases.

The neuronal semantic model incorporates features of iconic cortical neuronal circuits such as convergent projections, divergent projections, and lateral excitation. In addition, the model mimics processes occurring at the single neuron level that are based on the Hodgkin–Huxley model.

I simulated the convergent and divergent behavior of the model using an example English sentence. This model performed well in determining semantics of a complex sentence. The bottom-up is the feedforward and the top-down is the feedback projection of the neuronal network. The model simulates the binding and non-linear summation using the feedforward projection. The model then simulates the reactivation or accessing of the input neurons using the feedback projections.

## Background

This Background sections serves to highlight how the semantic network model and the MAYA semantic technique were engineered to mimic key features of cortical neuronal circuitry, neurophysiology and modern linguistic theory.

### The MAYA technique incorporates linguistic theory

A brief discussion of linguistic theory is important background to the MAYA Semantic Technique, because it operates on sentence structure. Human linguistic processing can be divided into separate modules that process different aspects of language (Embick et al., 2000, p. 6150) According to this theory, the syntactic module governs the structure of words and phrases in sentences. The MAYA technique functions as a syntactic module. The technique first determines categories and then identifies phrases. Then, based on this syntactic computation, the partial semantics of the sentence is computed. Sentence meaning is derived from syntactic structure and is dependent on the syntax for its combinatorial properties (Kuperberg, 2007, p. 24).

There is an important relationship between the predicate and the noun phrase in a sentence, as this structure is critical in human language. Predication has a one-to-one relationship in terms of the potential argument positions of a “predicate and the argument positions that are actually filled” (Smelser and Baltes, 2001, p. 15414), because English sentences have a verb noun repeating pattern within its syntactic structure. “Chomsky describes this one-to-one requirement between the predication position and the noun phrases filling this position as the theta-criterion” (Smelser and Baltes, 2001, p. 15414). This relationship is also expressed in the MAYA Semantic Technique. Within the MAYA Semantic Technique, summations are collected by special neurons. The summation is implemented to determine the number of verb noun repeating patterns within the sentence. A set of synchronized firings takes place and each special neuron reduces its count by 1 until the verb category (verb) is in its lowest term (v^1^). The reason that the MAYA Semantic Technique reduces each frequency count by 1 is that there is a one-to-one relationship between the verb and its object. This relationship is important in terms of the syntactic processing of sentences.

### The MAYA technique emulates key features of cortical neuronal circuits

I developed this model based on two related theoretical frameworks, whereby cortical circuits combine information from multiple sources (Byrne and Roberts, 2009, p. 526). The first framework is the convergence zone, which represents a many-to-one feedforward behavior. The sentence processing circuit of the anterior temporal cortex is considered to be a convergence zone (Damasio et al., 2004; Humphries et al., 2006; Jefferies and Lambon Ralph, 2006; Jefferies et al., 2008; Lau et al., 2008; Kiefer and Pulvermüller, 2012; Jefferies, 2013; Pulvermüller, 2013). The second framework is the divergence zone that represents a one-to-many feedback behavior of the neuronal circuit (Dayan and Abbott, 2005; Nicholls et al., 2011).

The MAYA frequency technique dynamically implements the convergent zone concept. It is modeled on a neuron's ability to non-linearly combine information received from several input neurons, in a compartmentalized gain modulation (Poirazi et al., 2003; Sarro, 2004; Spruston and Kath, 2004; Sidiropoulou et al., 2006). Each verb or noun phrase is mapped onto a single input neuron in the model network. In the frequency step, the information from all the different noun neurons (each representing an individual noun phrase in the sentence) converges onto one special neuron as a non-linear summation (this computes the total sum of noun phrases). The same computation is performed for verb phrases. This feedforward circuit is illustrated in more detail in the Methods Section that follows.

The MAYA reduction technique emulates the divergence zone. This technique is conceptually based on persistent action potential firing observed in some cortical neurons in the absence of stimulation (Sidiropoulou et al., 2006; Borzenko, 2010). In this step, the same special noun neuron that summated all the inputs, now fires bursts of spikes and activates all the input neurons representing different noun phrases in the sentence. Similarly the special verb diverges to activate multiple input verb neurons. This process reactivates the input neurons in the temporary neuronal network. More detailed explanation is provided in the Methods Section. Both the convergence and divergence projection of the neuronal network are parallel and sequential processes (Damasio, 1989, p. 36). Meaning is determined by the feedback reactivation of the input neurons (Damasio, 1989, p. 26).

### The MAYA technique emulates computational processes at the cellular level

The computational model implements the Hodgkin–Huxley model of neuronal computation at the cellular level. The neuronal computation is a non-linear computation. A computational model situated between the behavioral level and neurophysiological level was constructed to demonstrate the convergence and divergence projection of the neuronal network. The model is at the neurophysiological level because it models neurons and at the circuit level because it models the physiology of brain parts (Meeter et al., 2007).

## Method: the mathematical model derived from iconic neuronal circuits

The six processes of the MAYA Semantic Technique are shown below. For some sentences, the MAYA technique is able to compute other partial semantics that are specific only for those particular sentence structures (see the Supplementary Material II file). Thus, I developed a computer program that incorporates the six basic processes outlined below, in order to determine the partial semantics of sentences. The system includes a preprocessor that prepares the text, and it uses the part-of-speech tagger, parser, and name entity recognition software from the Stanford Natural Language Processing Group. In addition, I used the MontyLingua part-of-speech tagger from MIT.

1. The convergence behavior of the neuronal network is implemented mathematically using the **frequency technique** to compute partial semantics.
2. The divergence behavior of the neuronal network is implemented mathematically using the **reduction technique** to compute partial semantics.
3. The **full semantics** outputs the noun and prepositional phrase are outputted.
4. The **partial semantics I** outputs a shorter version of the full semantics.
5. The **partial semantics II** outputs a general overview of the sentence and it can be computed for some sentences.
6. The **partial semantic III** outputs a brief meaning of the sentence, which includes the main verb and its object.

**Processes 1 and 2 combine the Frequency and Reduction techniques of the MAYA Semantic Technique:**

The frequency technique is implemented first, followed by the reduction technique. To illustrate the method, the processes for computing semantics of an example sentence will be presented in detail with figures below.

Supplementary Table 1 shows the categories used by the MAYA Semantic Technique. Before each category is the ASCII code used to represent the categories during processing.

**Five categories and their associated colors used in the demonstration:**

The categories in the sentence are each represented by a different color. Each word in the sentence is given a number.

**Table:** 

noun	pronoun	verb	particle	preposition	conjunction
pink	pink	green	green	orange	blue

Many sentences are classified as complex sentences because they can be analyzed into a root sentence and at least one subordinate clause (Lyons, 1996).

Once a sentence has been broken down into categories, **Process 1 and 2** of the MAYA Semantic Technique is applied in six basic steps outlined below:

1. Determined the categories, and identify only the noun and verb phrases in the sentence.
2. Locate the first verb in the sentence.
3. Determine the subject of the sentence and words that may come before the first or main verb in the sentence.
4. Remove the subject from the sentence.
5. Determine the frequency of the categories in a non-linear summation.
6. Reduce the categories until the verb is in its lowest term (v^1^).

**The example sentence:**

*A 23 year old physics student has discovered an error in sir Isaac Newton's “Principia” that had gone undetected since the work laid out the laws of motion and gravity 300 years ago* (from The New York Times Corpus, Sandhaus, 2008).

In the semantic neuronal model, each word is mapped onto a different input word neuron in the network. Special neurons serve to compute information from multiple input word neurons. For example, the verb neuron receives input from three different neurons and integrates the inputs to produce an output that is the frequency of the verb categories. The equation below shows that the verb neuron has a count of three, meaning that the sentence has three verb neurons. It is possible that the special neuron achieves this computation using spatial summation.

$${\text{S}}_{0}={\text{v}}^{3}$$

Later, in the reduction technique the verb neuron reactivates three different neurons in the verb network to determine the main verb of the partial semantics. The equations below show the reduction of the verb neuron until it is in its lowest term in Equation S_2_.

$$\begin{array}{l} {\text{S}}_{0}={\text{v}}^{3}\\ {\text{S}}_{1}={\text{v}}^{2}\\ {\text{S}}_{2}={\text{v}}^{1} \end{array}$$

**Processes 1 and 2:**

**Step 1:** Determine the categories and identify the phrases in the sentence.

The MAYA Semantic Technique begins by grouping related categories. Hence, adjectives are grouped with nouns, and adverbs are grouped with verbs.

Each word in the sentence is represented as a neuron, as follows:

a 23 year old physics student has discovered an error in sir Isaac Newton principia that had gone undetected since the work laid out the laws of motion and gravity 300 years ago.

Each category in the sentence is also represented as a neuron, as follows:

art adject adject adject noun noun verb verb art noun prep noun art verb verb adject prep art noun verb adverb art noun prep noun conjun noun cardinal noun adverb.

The individual words and their categories are displayed in a table-like structure with numbers.

**Table:** 

1	2	3	4	5	6
a	23	year	old	physics	student
art	adject	adject	adject	noun	noun

**Figure 1:**
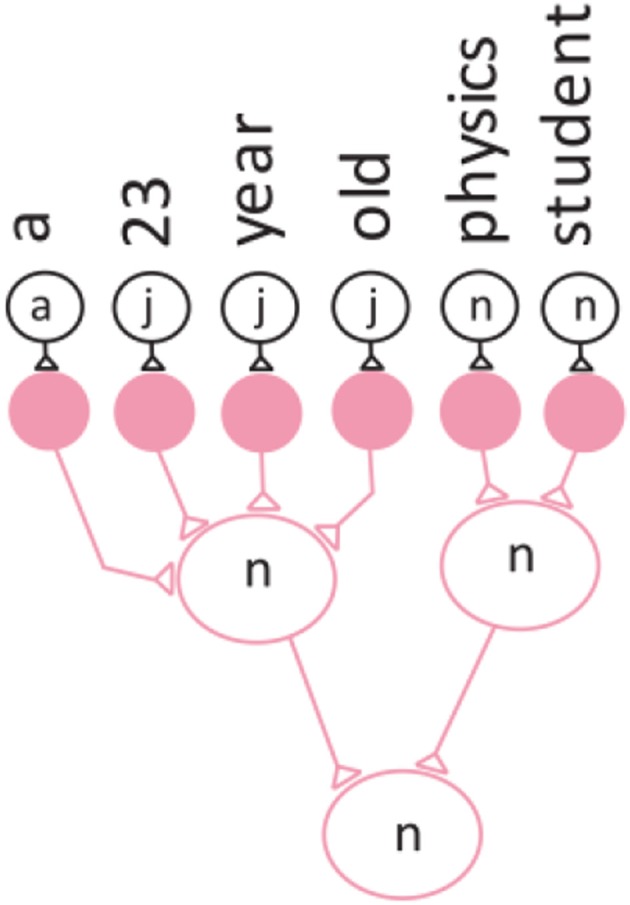
**Convergence projection of the neuronal network for a noun phrase**.

Figure 1 below shows how the words and categories are represented as a feed-forward iconic neuronal circuit, with each word mapped to a conceptual neuron in the circuit. The “art” neuron is stimulated by the “a” neuron in the network.

**Table:** 

1	2	3	4	5	6
a	23	year	old	physics	student
art	adject	adject	adject	noun	noun

**Table:** 

7	8
has	discovered
verb	verb

**Table:** 

9	10	11	12	13	14	15
an	error	in	sir	isaac	newton	principia
art	noun	prep	noun			

**Table:** 

16	17	18
that	had	gone
art	verb	verb

**Table:** 

19	20	21	22
undetected	since	the	work
adject	prep	art	noun

**Table:** 

23	24
laid	out
verb	adverb

**Table:** 

25	26	27	28	29	30	31
the	laws	of	motion	and	gravity	300
art	noun	prep	noun	conjun	noun	cardinal

**Table:** 

32	33
years	ago
noun	adverb

The neurons can concatenate adjacent words in a sentence in order to form phrases before the frequency of the category is determined, as shown here:

a-23-year-old-physics-student has-discovered an-error-in-sir-isaac-newton-principia that had-gone undetected-since-the-work laid-out the-laws-of-motion-and-gravity-300-years ago.

This grouping into phrases is achieved by the forward projection behavior of the convergence zone. Figure 1 illustrates the convergence projection behavior of the neuronal network.

Here the noun phrase “a-23-year-old-physics-student” is represented as a neuronal network. In this case the “noun” neuron receives input (stimulation) from six different neurons and integrates the inputs to produce an output.

Then, the frequency of each category is computed, reflecting the convergence of the objects. The frequency technique is used to determine how many verb and noun phrase patterns are in the sentence. The sentence is color-coded to show the categories and the phrases.

**Table:** 

1	2	3	4	5	6
a	23	year	old	physics	student
noun					

**Table:** 

7	8
has	discovered
verb	

**Table:** 

9	10	11	12	13	14	15
an	error	in	sir	isaac	newton	principia
noun						

**Table:** 

16	17	18
that	had	gone
art	verb	

**Table:** 

19	20	21	22
undetected	since	the	work
noun			

**Table:** 

23	24
laid	out
verb	

**Table:** 

25	26	27	28	29	30	31	32	33
the	laws	of	motion	and	gravity	300	years	ago
noun								adverb

Each category is represented by an ASCII character.

Sentence = noun verb noun verb noun verb noun.

**Figure 2:**
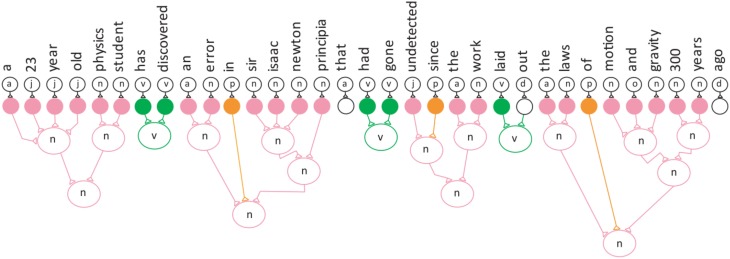
**The semantic neuronal network for the example sentence**.

The categories are represented by Equation (1). Figure 2 depicts the entire model semantic neuronal network with all its verb and noun phrases.

(1) S=n+v+n+v+n+v+n

The equation for the entire semantic neuronal network.

**Step 2:** Locate the first verb, “has-discovered,” in the sentence.
**Step 3:** Determine the subject of the sentence and the words preceding the first verb in the sentence.
                   **Subject** = a-23-year-old-physics-student.
**Step 4:** Remove the subject from the sentence.

**Figure 3:**
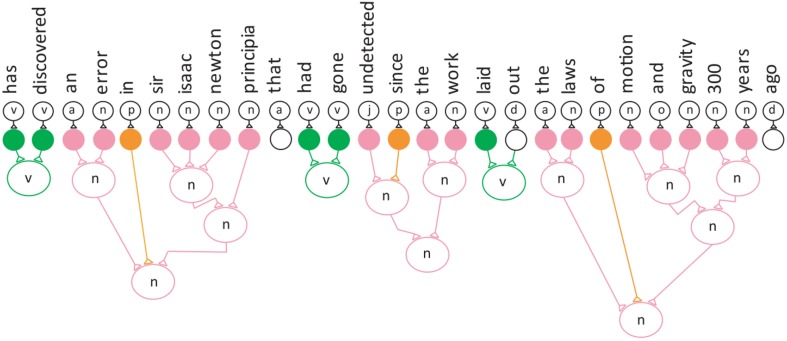
**Semantic neuronal network after Step 4**.

Equation (2) and Figure 3 represent the semantic neuronal network after the removal of the subject.

(2) S=v+n+v+n+v+n

The semantic neuronal network after Step 4.

Next, steps 5 and 6 of the MAYA Semantic Technique will be discussed in detail along with the conceptual model of the verb neuronal network.

**Figure 4:**
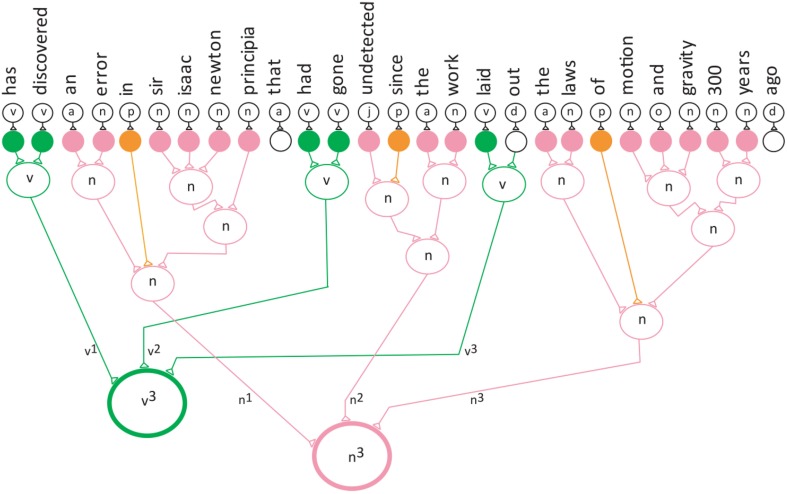
**Convergence projection of the neuronal network for the verb and noun phrase**.

**Step 5:** The frequency technique determines the number of verb and noun phrase patterns in a sentence. This step uses non-linear summation modeled after neuronal behavior (Segev and Rail, 1998). The categories can be thought of as analogous to neuronal inputs to different dendrites that are summed. To compute the non-linear summation of categories the neurons in the network must fire simultaneously to their special neuron. After all the neurons in the network fire simultaneously, the non-linear spatial summation takes place. Step 5 of the MAYA Semantic Technique incorporates both the simultaneous network bursting and the spatial summation behavior observed in neurons the brain. Network bursting is when many neurons in a neuronal network all fire simultaneously followed by a period of quiescence (Thomas et al., 1999; Izhikevich, 2010). In the model network, the special neurons use spatial summation in the form of simultaneous addition from multiple neurons (Byrne and Roberts, 2009, p. 487). Equation (3) expresses the frequency of the verb and noun and Figure 4 depicts the semantic neuronal network for step 5.

(3) $${\text{S}}_{0}={\text{v}}^{3}+{\text{n}}^{3}$$

The frequency for both the verb and noun phrase.

The indicators v^3^ (verb) and n^3^ (noun) show that there are three verbs in the verb network and three nouns in the noun network. Figure 4 demonstrates a convergence projection for both the noun and verb networks. The verb^3^ and noun^3^ neuron are special neurons that are stimulated by three different neurons and keep track of the sum of the individual categories.

**Step 6:** The reduction technique is used to identify the verb and noun phrase groups within the semantic neuronal network. After all of the summations are collected by the special neurons, another set of synchronized firing takes place. Each special neuron reduces its count by 1 until the verb category (verb) is in its lowest term (v^1^). In the model each reduction that takes place is analogous to an action potential. Thus, multiple reductions result in repetitive firing of the special neuron. The frequency for each category is reduced by 1. It is important to note that the special neuron, in this case, verb^3^, points to the third neuron in the sentence and processes the sentence in the reverse order (from the end of the sentence to the beginning of the sentence) in order to determine the main verb in the sentence. At the completion of the reduction technique, the special neuron projects to the main verb in the sentence.

The reduction technique is a variation of the derivative in calculus.

$$\frac{d}{dx}x^{y}=nx^{y-1}$$

The coefficient *n* is not represented in the equation.

Supplementary Table 1 shows the categories used by the MAYA Semantic Technique. Before each category is the ASCII code used to represent the categories during processing.

The reduction technique is as follows:

$$S_{0}\;=v^{t}+\;n^{w}$$

S_0_ is the structure of the sentence under investigation, where n and v, represent the categories making up S_0_, and *t* and *w*, represent the count of each category of S_0_. S_1_ is the first reduction of the sentence S_0_, which is derived by reducing each count of each category by 1.

$$If\{v^{e}\}=1,\;thenstop,\;else\{v^{e-1}\}$$

Subtract 1 from each count until the count of the verb category is one.

$$\begin{array}{l} S_{1}\;=v^{t-1}+\;n^{w-1}\\ S_{y}=v^{t-y}+\;n^{w-y} \end{array}$$

The letter *t* represents the highest count of the category “verb” Therefore, S_*y*_ is the final reduction of sentence S_0_.

Here is an example of a sentence being reduced:

$$\begin{array}{l} {\text{S}}_{0}={\text{verb}}^{4}+{\text{noun}}^{4}\\ {\text{S}}_{1}={\text{verb}}^{3}+{\text{noun}}^{3}\\ {\text{S}}_{2}={\text{verb}}^{2}+{\text{noun}}^{2}\\ {\text{S}}_{3}={\text{verb}}^{1}+{\text{noun}}^{1} \end{array}$$

**Figure 5:**
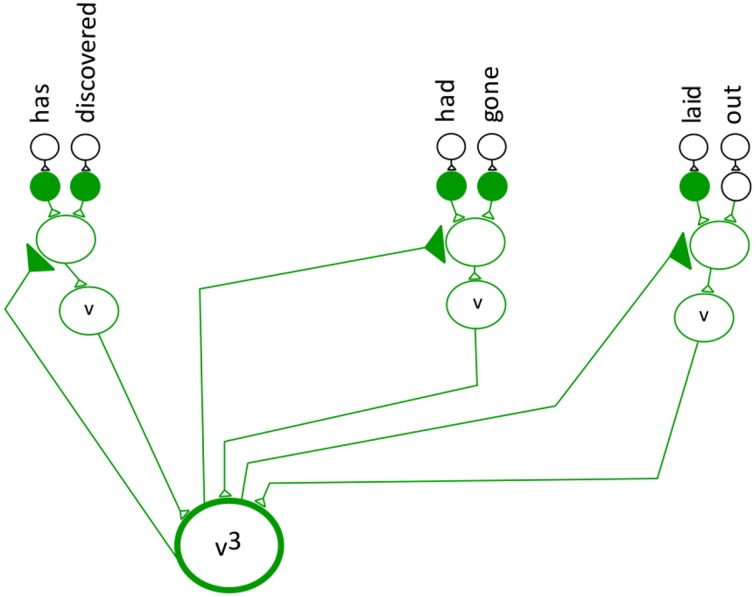
**Convergence/divergence projection behavior of the neuronal network for the verb phrase**.

The computation was developed based on the conceptual model of the verb network shown in Figure 5. Here the verb^3^ neuron is stimulated by three different verb neurons (convergence) and the verb^3^ neuron stimulates the three different neurons (divergence).

To show the feedback to the input neurons an additional layer was added to the divergence projection neuronal network. This is the first demonstration of semantic neuronal network with an added layer of divergence.

### Simulating the reduction technique on the verb phrase network

**Figure 6:**
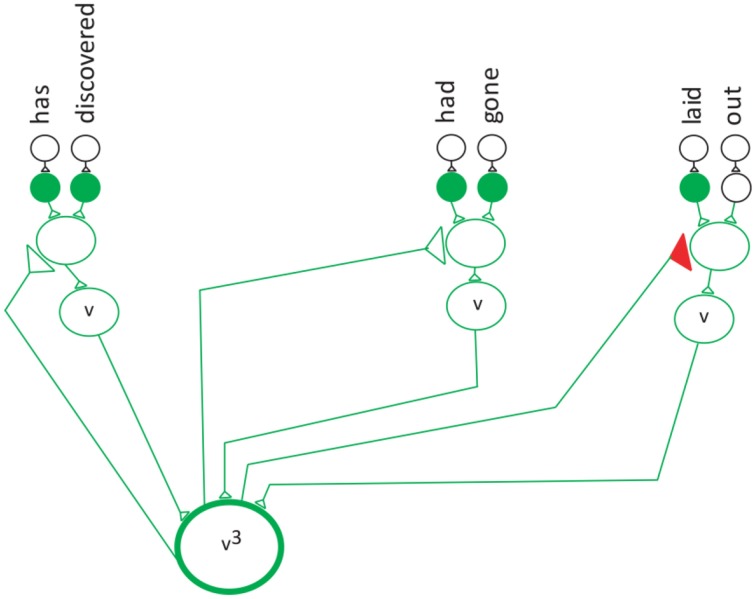
**The verb neuron starts the reduction**.

The next three figures further illustrate the reduction technique. Figure 6 represents a divergence projection of the neuronal network that shows the special neuron projecting to the last neuron (the third verb) in the verb network, which points to the words “laid-out.” The next step in the reduction technique is to reduce each of the frequencies by 1.

(4) $$S_{0}=v^{3}+n^{3}$$

The frequency for both the verb and noun phrase.

(5) $$S_{1}=v^{2}+n^{2}$$

The first reduction. S_1_ denotes the first reduction occurred.

**Figure 7:**
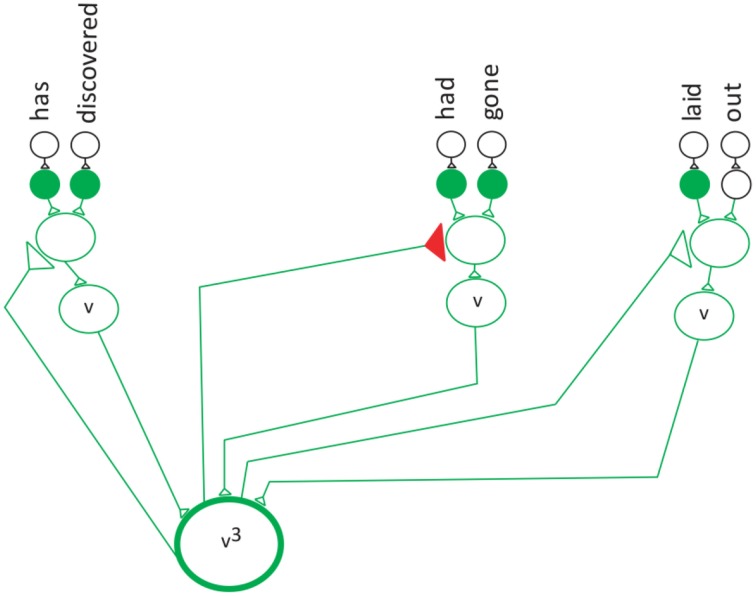
**The verb neuron after the first reduction**.

**Figure 8:**
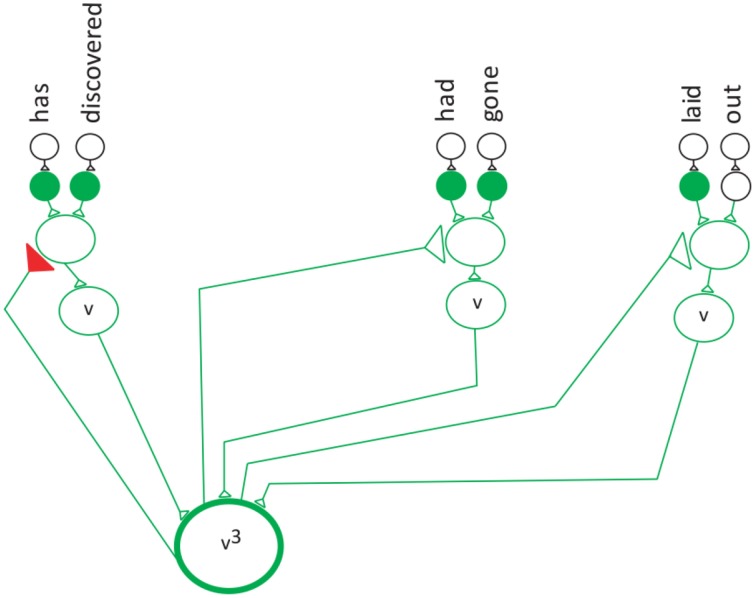
**The verb neuron after the second reduction**.

S_1_ means that the first reduction has taken place. In the first reduction step the frequency of each category is reduced by one (Equation 5, Figure 7). The v^2^ and n^2^ indicates that the second verb and the second noun in the sentence are being pointed to by their special neurons. Figure 8 shows that verb^2^ fires and that its count is reduced by 1. Then verb^2^ projects to the second neuron in the verb network, which projects to the verb with the label “had-gone.” This is followed by the second reduction in which verb^1^ fires reduces the category count by 1 (Equation 6, Figure 8). The S_2_ cannot be reduced further, because the category v^1^ is in its lowest terms. The verb^1^ neuron projects to the first neuron in the verb network, which has the label “has-discovered.”

(6) $${\text{S}}_{2}={\text{v}}^{1}+{\text{n}}^{1}$$

The second reduction.

Equation (6) shows that the first verb and the first noun are the verb and its object in the sentence.

Equation (6) and Figure 8 show the special neuron pointing to the first verb phrase.

Table 1 shows the verb and noun phrase group for the sentence, where **S = v^3^ + n^3^** points to the last verb and noun phrase group in the sentence.

**Table 1:** **The verb and noun phrase patterns in the sentence**.

**The reduction**	**The corresponding words for the verb and noun phrase**
S_0_ = v^3^ + n^3^	Laid out the laws of motion and gravity 300 years ago
S_1_ = v^2^ + n^2^	That had gone undetected since the work
S_2_ = v^1^ + n^1^	Has discovered an error in sir Isaac Newton principia

**Figure 9:**
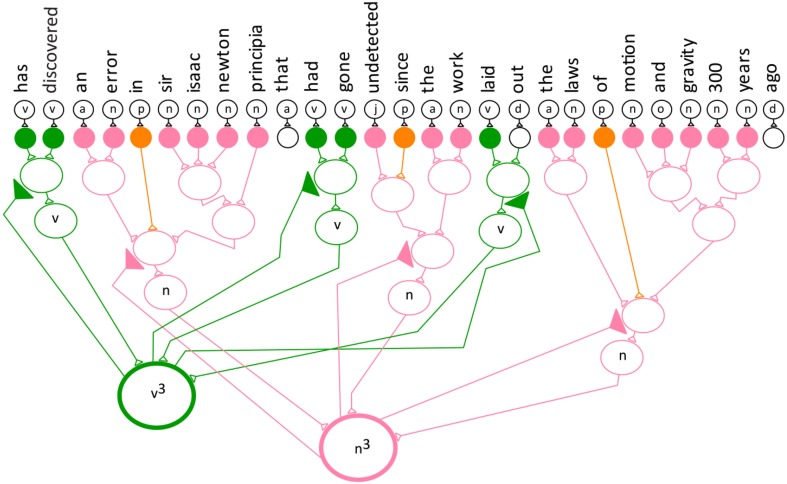
**The divergence projection of the neuronal network for the entire sentence**.

Figure 9 shows the divergence projection of the neuronal network for the entire sentence. Each time the reduction technique executes, it points to a different verb and noun phrase group in the sentence.

The reduction technique implements the feedback projection within the network, and as a result, it reactivates the words grouped into verb and noun phrases. The sentence is parsed into several verb and noun phrase groups, since English sentences have a repeating pattern of verb-and-noun phrase. The divergence projection of the semantic neuronal network identifies the main verb phrase in the sentence.

(7) $$\text{S}={\text{v}}^{1}+{\text{n}}^{1}+{\text{v}}^{2}+{\text{n}}^{2}+{\text{v}}^{3}+{\text{n}}^{3}$$

The general equation.

In Equation 7, **v^3^ + n^3^** represents the third verb and noun phrase group in the sentence.

The general equation of the sentence is used to determine the full semantics, partial semantics I, and partial semantics III.

**Process 3: The full semantic**

The general equation for the full semantics of the sentence is as follows:

(8) $$\text{The full semantics}={\text{V}}^{1}+{\text{N}}^{1}+{\text{V}}^{2}+{\text{N}}^{2}+{\text{V}}^{3}+{\text{N}}^{3}$$

V^3^ + N^3^ represents the third position within the sentence.

**Figure 10:**
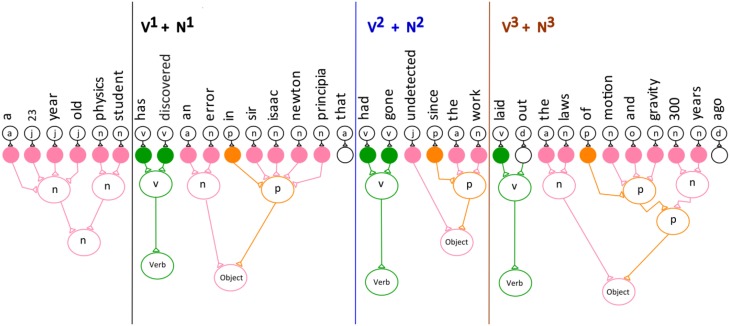
**The network model for the full semantics of the entire sentence with its verb and object patterns**.

Within each Verb Noun group the verb phrase and the noun phrase and/or prepositional phrase are determined. To determine the full semantics of the sentence the noun phrase must be changed into noun and prepositional phrases. The object typically follows the verb phrase. If the first phrase after the verb phrase is a noun phrase then it will be the object. If a prepositional phrase also follows the noun phrase, then it will also will be included as part of the object. The noun phrase (np) and the prepositional phrase (pp) will represent the object for that group. Figure 10 depicts the entire sentence with its full semantics. Each verb and noun phrase group represents a verb and its object. The verbs and their object patterns are listed in Supplementary Table 2. Adjectives are changed to nouns in order to determine the object.

The verb and noun group patterns along with the corresponding words for the sentence are listed in Supplementary Table 3.

The output of the full semantic process, shown below, is almost an exact reproduction of the original sentence except for a few details.

a 23 year old physics student has discovered an error in sir Isaac Newton principia had gone undetected since the work laid out the laws of motion and gravity 300 years.

**Process 4: The partial semantics I**

**Figure 11:**
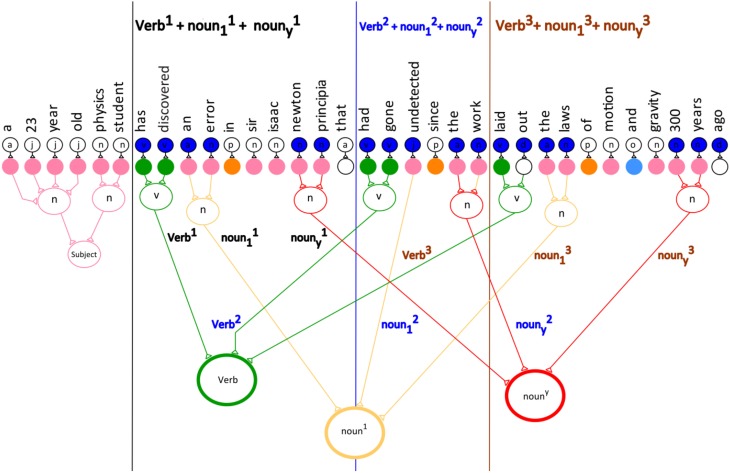
**The network model for the partial semantics I for the example sentence**.

The partial semantics I process produces a slightly smaller version of the sentence than the full semantics. The verb, as well as the first and the last noun phrase within each verb-noun phrase group are computed. The network processing in partial semantics I is analogous to lateral excitation. Lateral excitation is implemented to determine the meaning of the sentence. Figure 11 shows the verb and noun phrases that are included in the partial semantics I for the sentence.

The general equation for the partial semantics I is:

$$\begin{array}{l} \text{Partial Semantic I}=Verb^{1}+noun_{1}^{1}+noun_{y}^{1}+Verb^{2}+noun_{1}^{2}\\ \;+noun_{y}^{2}+Verb^{3}+noun_{1}^{3}+noun_{y}^{3}\\ \;+\ldots Verb^{m}+noun_{1}^{m}+noun_{y}^{m} \end{array}$$

where $Verb^{3}+noun_{1}^{3}+noun_{y}^{3}$ represents the third verb and noun phrase group in the sentence.

Specifically, $noun_{1}^{3}+noun_{y}^{3}$ denotes the first noun and the last noun, respectively in the third verb and noun phrase group within the sentence.

The partial semantics I for the sentence is:

$$\begin{array}{l} \text{Partial Semantic I}=Verb^{1}+noun_{1}^{1}+noun_{y}^{1}+Verb^{2}+noun_{1}^{2}\\ \;+noun_{y}^{2}+Verb^{3}+noun_{1}^{3}+noun_{y}^{3} \end{array}$$

Supplementary Table 4 shows a detailed equations for each verb and noun phrase group within the sentence.

The partial semantics I for the sentence is:

a 23 year old physic student has discovered an error Newton principia had gone undetected the work laid out the laws 300 years.

Table 2 illustrates the subject with the first verb and its noun phrase pattern. $Verb^{1}$ is the first and main verb in the sentence. The first noun in the noun phrase is $noun_{1}^{1}$ and the last noun in the phrase is $noun_{y}^{1}$.

**Table 2:** **The subject with the verb and its first and last noun phrase**.

**Subject**	**Verb^1^**	***noun*^1^_1_**	***noun*^1^_y_**
A 23 year old physic student	Has discovered	An error	Newton principia

Figure 11 shows the partial semantics I for the sentence where the $Verb+noun_{1}+noun_{y}^{1}$ pattern is computed.

**Process 6: The partial semantic III**

The partial semantics III computes a brief meaning of the sentence that includes the main verb and its object. This is illustrated in Table 3.

**Table 3:** **The partial semantic III**.

**Subject**	**Verb**	**Object/Complement**
**A-23-year-old-physics-student**	**Has-discovered**	**An-error-in-sir-Isaac-Newton-principia**

**Figure 12:**
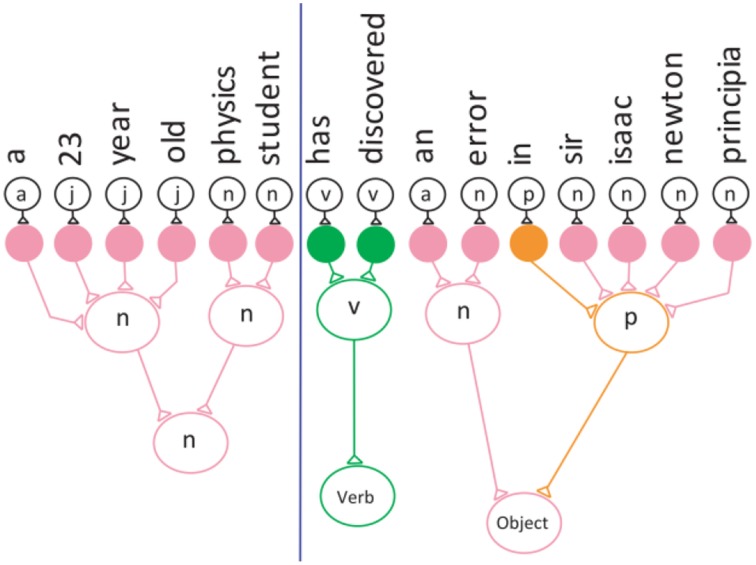
**The partial semantics III network model for part of the example sentence**.

Figure 12 depicts the network model of the subject, verb, and object for the sentence. The object is composed of a noun and a prepositional phrase.

In processes 3 through 6, lateral excitation is used to determine partial semantics. Semantic processing for English sentences is executed from left to right. As a result, the lateral excitations are processed from left to right within the sentence.

## Results

A simulation of semantic processing of the example sentence in a model network.

Computational models were constructed from the conceptual model in Figure 9. A Hodgkin–Huxley network model was used to demonstrate the verb network for the sentence above.

The conceptual model in Figure 9 was used to develop two computational models that simulate the steps in the MAYA Semantic Technique. Two different simulations of the verb network were performed in order to show how the neurons in the cortex might execute a small segment of the example sentence. The first neuronal model simulates the convergence projection of the verb and noun neuronal network for the frequency of the neurons. The second neuronal model simulates the divergence projection of the verb neuronal network (step 6 of the MAYA Semantic Technique). The default values from the Simulator for Neuronal Networks and Action Potentials (SNNAP) software from the University of Texas Health Science Center was used for the network.

### Simulation I of the convergence projection behavior of the semantic neuronal network

I simulated the frequency technique of the MAYA Semantic Technique in which the non-linear summation takes place on the categories. The conceptual model is shown in Figure 9. Supplementary Table 5 displays the labels used in the simulation. Supplementary Table 6 shows each neuron with an duration of 3 ms and a current injection stimulation value of 5 μA/cm^2^.

Supplementary Figure 1 shows that an action potential for the verb and noun neurons occurred at about 3 ms, and, that at approximately 11 ms, it approached the resting potential of −60 mV. Because it is a convergence projection of the neuronal network, all the input neurons execute in parallel (simultaneously), giving the “Verb” neuron a numeric value of three. The noun network in Supplementary Figure 1 has the same values as the verb network. Therefore, the neurons in both the verb and noun networks in Supplementary Figure 1 fired simultaneously followed by a period of quiescence. Supplementary Figure 1 shows the parallel execution of the three input neurons to produce a single action potential, the non-linear summation for the verb and noun categories. It is theorized that the verb and noun neurons with the value of three may produce an action potential in order to point to the last verb in the verb network based on the frequency value because the next step requires the verb neuron to execute the reduction technique. The reduction technique requires the verb neuron to point to the last verb in the sentence in order to successfully implement Step 6 of the MAYA Semantic Technique.

### Simulation II of the divergence projection behavior of the verb neuronal network

I simulated the reduction technique of the MAYA Semantic Technique. The reduction technique reduces each category by one until the verb category is in its lowest term. The conceptual model is shown in Figure 6. Supplementary Table 7 shows that the firing of the neuron has a duration of 3 ms, and each neuron starts 1 ms after the previous neuron, with a current injection stimulation value of 5 μA/cm^2^. Supplementary Figure 2 shows that an action potential for the first input neuron occurred at about 3 ms, and that at approximately 13 ms, it approached the resting potential of −60 mV. At the beginning of the processing, the “verb” neuron projects to verb “laid out” (see Figure 7). At the end of the processing, the “verb” neuron projects to the main verb in the sentence, which is “has-discovered” (see Figure 8).

Supplementary Figure 2 shows how the verb neuron produces three action potentials 1 ms apart. This simulates the reduction technique, which equates to the firing of the neuron in the absence of stimulation.

While working on the neuroscience research I realized I had to modify the original mathematical technique. The original mathematical technique grouped the categories into several phrases such as verb, noun, and prepositional phrase into an equation. I realize that I had to group the categories into only two phrases: verb and noun, since it was the repeating pattern in English sentences.

Another finding of this neuroscience research was the lateral excitation which took place after the reduction technique executed. I found that during the lateral excitation the neuronal network might select the phrases that will be included in the partial semantics. Hence, this current research found three partial semantics that might be computed by the neuronal network. The three partial semantics includes the full, partial semantic I, partial semantic II (see the Supplementary Material II file), and the partial semantic III. These partial semantics have not been implemented within a natural language processing program.

## Discussion

Cognitive scientists have begun to formulate mechanistic accounts of how language is processed in the brain (MacWhinney and Ping, 2008). MacWhinney and Ping (2008, p. 234) discussed a syntactic emergence model of “ambiguity resolution in sentence processing that is grounded on competition between lexical items.” They noted that these syntactic emergent models are able to model the temporal properties of sentence processing, but these do not decode the basic addressing system of the brain (MacWhinney and Ping, 2008, pp. 234–235). The simulation of a neuronal network in this paper demonstrates detail processing, where each word in a sentence is represented by a neuron implementing integrate-and-fire techniques.

Pulvermüller (2013) presented a model of language processing at a macroscopic level within a distributed neuronal networks. This research demonstrated sentence processing at a microscopic level. The simulation of the partial semantics of a sentence demonstrated how the mathematical techniques was represented at a microscopic level where each word was represented as a neuron.

My model emulates features of neuronal language processing both at the circuit and cellular level. The feedback and feedforward projections of the network emulate circuit behavior. The neuronal summation mimics cellular behavior. The feedback projection can reactivate the input neurons by a reduction technique as described by the MAYA Semantic Technique. Interestingly, the feedback behavior of the model network may also be a feature of the neural correlates of consciousness. Frontoparietal feedback connectivity was dramatically affected by either propofol treatment or general anesthesia (Ku et al., 2011, p. 3).

In the semantic neuronal network model, binding takes place when the neurons in the network all fire simultaneously. The forward projection behavior of the convergence zone groups the categories into phrases. Then the frequency of each category is computed which reflects the convergence of the objects. The frequency technique is used to determine how many verb and noun phrase patterns are in the sentence.

The network might also use feedback projections to in order to select one input neuron at the exclusion of the others. Although not present in the current network, inhibitory neurons would be crucial for this selection task, because they could block or exclude certain parts of the network. Thus, these signals would be excluded from attention during the feedback propagation (Strüber et al., 2015, p. 1). It is theorized that during the selection or feedback projection, neurons exhibit a cascading behavior to select parts of the network to be included in attention. Thus, the network model shares features with neuronal circuit behavior important for attention.

Future research is needed to demonstrate how the brain comprehends sentences within a distributed semantic neuronal network that implements lexical relations among the verb and noun phrases in the sentence. A larger goal is to determine how the brain comprehends more complex text, such as news articles, which would involve multiple semantic neuronal networks.

### Conflict of interest statement

The author declares that the research was conducted in the absence of any commercial or financial relationships that could be construed as a potential conflict of interest.

## References

[B1] Awad M. Warren J. E. Scott S. K. Turkheimer F. E. Wise R. J. S. (2007). A common system for the comprehension and production of narrative speech. J. Neurosci. 27, 11455–11464. 10.1523/JNEUROSCI.5257-06.2007 17959788 PMC6673222

[B2] Borzenko A. (2010). A neural mechanism for human language processing. Neurocomputing 74, 104–112. 10.1016/j.neucom.2009.11.051

[B3] Byrne J. H. Roberts J. L. (eds.). (2009). From Molecules to Networks: An Introduction to Cellular and Molecular Neuroscience, 2nd Edn Burlington, MA: Academic Press.

[B5] Christensen K. R. (2008). Interfaces, syntactic movement, and neural activation: a new perspective on the implementation of language in the brain. J. Neurolinguist. 21, 73–103. 10.1016/j.jneuroling.2007.01.002

[B6] Cooke A. DeVita C. Gee J. Alsop D. Detre J. Chen W.. (2003). Neural basis for sentence comprehension deficits in frontotemporal dementia. Brain Lang. 85, 211–221. 10.1016/S0093-934X(02)00562-X 12735939

[B7] Damasio A. R. (1989). Time-locked multiregional retroactivation: a systems-level proposal for the neural substrates of recall and recognition. Cognition 33, 25–62. 10.1016/0010-0277(89)90005-X 2691184

[B8] Damasio H. Tranel D. Grabowski T. Adolphs R. Damasio A. (2004). Neural systems behind word and concept retrieval. Cognition 92, 179–229. 10.1016/j.cognition.2002.07.001 15037130

[B9] Dayan P. Abbott L. F. (2005). Theoretical Neuroscience: Computational and Mathematical Modeling of Neural Systems. Cambridge, MA: MIT Press.

[B10] Dronkers N. F. Wilkins D. P. van Valin R. D. Jr. Redfern B. B. Jaeger J. J. (2004). Lesion analysis of the brain areas involved in language comprehension. Cognition 92, 145–177. 10.1016/j.cognition.2003.11.002 15037129

[B11] Embick D. Marantz A. Miyashita Y. O'Neil W. Sakai K. L. (2000). A syntactic specialization for Broca's area. Proc. Natl. Acad. Sci. U.S.A. 97, 6150–6154. 10.1073/pnas.100098897 10811887 PMC18573

[B12] Fell J. Axmacher N. (2011). The role of phase synchronization in memory processes. Nat. Rev. Neurosci. 12, 105–118. 10.1038/nrn2979 21248789

[B13] Humphries C. Binder J. R. Medler D. A. Liebenthal E. (2006). Syntactic and semantic modulation of neural activity during auditory sentence comprehension. J. Cogn. Neurosci. 18, 665–679. 10.1162/jocn.2006.18.4.665 16768368 PMC1635792

[B14] Izhikevich E. M. (2010). Dynamical Systems in Neuroscience: The Geometry of Excitability and Bursting. Cambridge, MA: MIT Press.

[B15] Jefferies E. (2013). The neural basis of semantic cognition: converging evidence from neuropsychology, neuroimaging and TMS. Cortex 49, 611–625. 10.1016/j.cortex.2012.10.008 23260615

[B16] Jefferies E. Hoffman P. Jones R. Lambon Ralph M. A. (2008). The impact of semantic impairment on verbal short-term memory in stroke aphasia and semantic dementia: a comparative study. J. Mem. Lang. 58, 66–87. 10.1016/j.jml.2007.06.004 18438454 PMC2344152

[B17] Jefferies E. Lambon Ralph M. A. (2006). Semantic impairment in stroke aphasia versus semantic dementia: a case-series comparison. Brain 129, 2132–2147. 10.1093/brain/awl153 16815878

[B18] Jurafsky D. Martin J. H. (2008). Speech and Language Processing, 2nd Edn Upper Saddle River, NJ: Prentice Hall.

[B19] Kiefer M. Pulvermüller F. (2012). Conceptual representations in mind and brain: theoretical developments, current evidence and future directions. Cortex 48, 805–825. 10.1016/j.cortex.2011.04.006 21621764

[B20] Ku S.-W. Lee U. Noh G.-J. Jun I.-G. Mashour G. A. (2011). Preferential inhibition of frontal-to-parietal feedback connectivity is a neurophysiologic correlate of general anesthesia in surgical patients. PLoS ONE 6:e25155. 10.1371/journal.pone.0025155 21998638 PMC3187752

[B21] Kuperberg G. R. (2007). Neural mechanisms of language comprehension: challenges to syntax. Brain Res. 1146, 23–49. 10.1016/j.brainres.2006.12.063 17400197

[B22] Lau E. F. Phillips C. Poeppel D. (2008). A cortical network for semantics: (De)constructing the N400. Nat. Rev. Neurosci. 9, 920–933. 10.1038/nrn2532 19020511

[B23] Lyons J. (1996). Linguistic Semantics: An Introduction. Cambridge: Cambridge University Press.

[B24] MacWhinney B. Ping L. (2008). Neurolinguistic computational models in Handbook of the Neuroscience of Language, 1st Edn, eds. Stemmer B. Whitaker H. A. (London: Academic Press), 229–236. 10.1016/B978-0-08-045352-1.00022-7

[B25] Meeter M. Jehee J. Murre J. (2007). Neural models that convince: model hierarchies and other strategies to bridge the gap between behavior and the brain. Philos. Psychol. 20749–20772. 10.1080/09515080701694128

[B26] Nicholls J. G. Martin A. R. Fuchs P. A. Brown D. A. Diamond M. E. Weisblat D. (2011). From Neuron to Brain, 5th Edn Sunderland, MA: Sinauer Associates.

[B27] Poirazi P. Brannon T. Mel B. W. (2003). Arithmetic of subthreshold synaptic summation in a model CA1 pyramidal cell, Neuron 37, 977–987. 10.1016/S0896-6273(03)00148-X 12670426

[B28] Pollack M. E. (2005). Intelligent technology for an aging population: The use of AI to assist elders with cognitive impairment. AI Magazine 26, 9–24. 10.1609/aimag.v26i2.1810

[B29] Pulvermüller F. (2013). How neurons make meaning: brain mechanisms for embodied and abstract-symbolic semantics. Trends Cogn. Sci. 17, 458–470. 10.1016/j.tics.2013.06.004 23932069

[B30] Pulvermüller F. Fadiga L. (2010). Active perception: sensorimotor circuits as a cortical basis for language. Nat. Rev. Neurosci. 11, 351 10.1038/nrn2811 20383203

[B31] Russell S. Norvig P. (2009). Artificial Intelligence: A Modern Approach, 3rd Edn Upper Saddle River, NJ: Prentice Hall.

[B32] Sandhaus E. (2008). The New York Times Annotated Corpus. Philadelphia, PA: Linguistic Data Consortium.

[B33] Sarro L. M. (2004). Characterization of dendrites as nonlinear computation devices. Neurocomputing 58–60, 581–586. 10.1016/j.neucom.2004.01.098

[B34] Schirmer A. (2004). Timing speech: a review of lesion and neuroimaging findings. Cogn. Brain Res. 21, 269–287. 10.1016/j.cogbrainres.2004.04.003 15464357

[B35] Segev I. Rail W. (1998). Excitable dendrites and spines: earlier theoretical insights elucidate recent direct observations. Trends Neurosci. 21, 453–460. 10.1016/S0166-2236(98)01327-7 9829684

[B36] Sidiropoulou K. Pissadaki E. K. Poirazi P. (2006). Inside the brain of a neuron. EMBO Rep. 7, 886–892. 10.1038/sj.embor.7400789 16953202 PMC1559659

[B37] Smelser N. J. Baltes P. B. (2001). International Encyclopedia of the Social & Behavioral Sciences. Oxford: Elsevier.

[B38] Sonty S. P. Mesulam M. M. Weintraub S. Johnson N. A. Parrish T. B. Gitelman D. R. (2007). Altered effective connectivity within the language network in primary progressive aphasia. J. Neurosci. 27, 1334–1345. 10.1523/JNEUROSCI.4127-06.2007 17287508 PMC6673590

[B39] Spruston N. Kath W. L. (2004). Dendritic arithmetic. Nat. Neurosci. 7, 567–569. 10.1038/nn0604-567 15162161

[B40] Strüber M. Jonas P. Bartos M. (2015). Strength and duration of perisomatic GABAergic inhibition depend on distance between synaptically connected cells. Proc. Natl. Acad. Sci. U.S.A. 112, 1220–1225. 10.1073/pnas.1412996112 25583495 PMC4313861

[B41] Thomas E. A. Bertrand P. P. Bornstein J. C. (1999). Genesis and role of coordinated firing in a feedforward network: a model study of the enteric nervous system. Neuroscience 93, 1525–1537. 10.1016/S0306-4522(99)00243-2 10501477

[B42] Ullman M. T. (2001). A neurocognitive perspective on language: the declarative/procedural model. Nat. Rev. Neurosci. 2, 717. 10.1038/35094573 11584309

[B43] von Neumann J. (2000). The Computer and the Brain, 2nd Edn New Haven, CT: Yale University Press.

[B44] Wright P. Stamatakis E. A. Tyler L. K. (2012). Differentiating hemispheric contributions to syntax and semantics in patients with left-hemisphere lesions. J. Neurosci. 32, 8149–8157. 10.1523/JNEUROSCI.0485-12.2012 22699896 PMC3575031

