# Supplementary material for: Semantic processing of English sentences using statistical computation based on neurophysiological models
Source: Front Physiol. 2015 May 22;6:135. doi: 10.3389/fphys.2015.00135 (PMC4460779; doi:10.3389/fphys.2015.00135)
Supplement: Supplementary file 1 [file Presentation1.PDF]

Semantic processing of English sentences using statistical computation based on neurophysiological models

Marcia T. Mitchell

Computer and Information Sciences Department, Saint Peter's University, Jersey City, New Jersey, USA, mmitchell@saintpeters.edu

Supplementary Figures and Tables

a = article	d = adverb	h = prepositional phrase
J = adjective	n = noun or noun phrase	o = conjunction
p = preposition/particle	q = cardinal	r = pronoun
	v = verb or verb phrase	

Supplementary Table 1. The categories in the sentences.

**General equation**	**The specific equation for each verb and noun phrase group**
$V^1 + N^1$	Verb + Noun Phrase + Prepositional Phrase
$V^2 + N^2$	Verb + Noun + Prepositional Phase
$V^3 + N^3$	Verb + Noun + Prepositional Phase

Supplementary Table 2. The verb and its object for the full semantics.

**General equation**	**The corresponding words for the equation**
$V^1 + N^1$	has discovered an error in sir isaac newton principia
$V^2 + N^2$	had gone undetected since the work
$V^3 + N^3$	laid out the laws of motion and gravity 300 years ago

Supplementary Table 3. The actual phrases that make up the full semantic.

**The specific equation**	**The corresponding words for the equation**
$\text{Verb}^1 + noun_1^1 + noun_y^1$	has discovered an error newton principia
$\text{Verb}^2 + noun_1^2 + noun_y^2$	had gone undetected the work
$\text{Verb}^3 + noun_1^3 + noun_y^3$	laid out the laws 300 years

Supplementary Table 4. The phrases associated with the partial semantics I.

**Verb Network**	**Noun Network**
has discovered (verb1)	an error in sir isaac newton principia (noun1)
had gone (verb2)	undetected since the work (noun2)
laid out (verb3)	the laws of motion and gravity 300 years (noun3)

Supplementary Table 5. The verb and noun phrases with corresponding labels used in the simulation.

**Neuron**	**Start Time**	**Stop Time**	**Magnitude**
verb1 (has discovered)	0.000	0.003	5.00
verb2 (had gone)	0.000	0.003	5.00
verb3 (laid out)	0.000	0.003	5.00
Verb	0.003	0.006	5.00

Supplementary Table 6. The treatment of the verb network.

**Neuron**	**Start Time**	**Stop Time**	**Magnitude**
verb3 (laid-out)	0.000	0.003	5.00
verb2 (had-gone)	0.001	0.004	5.00
verb1 (has-discovered)	0.002	0.005	5.00

Supplementary Table 7. The second treatment of the verb network.

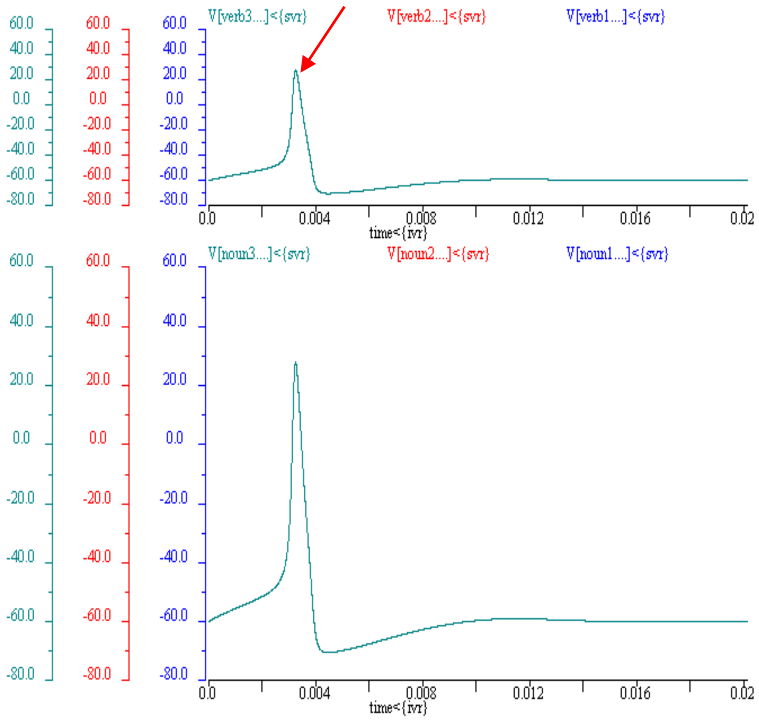

Supplementary Figure 1. Simulation of the verb and noun phrase network from Figure 4. The arrow points to the verb frequency.

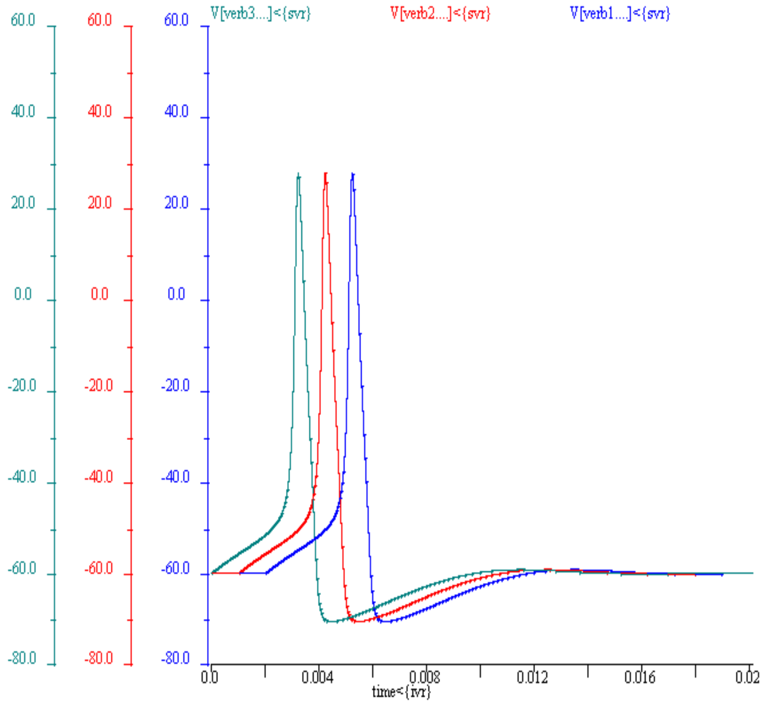

Supplementary Figure 2. Simulation of the divergence projection of the verb neuronal network.
