# Supplementary material for: Semantic processing of English sentences using statistical computation based on neurophysiological models
Source: Front Physiol. 2015 May 22;6:135. doi: 10.3389/fphys.2015.00135 (PMC4460779; doi:10.3389/fphys.2015.00135)
Supplement: Supplementary file 2 [file Presentation2.PDF]

Supplementary Material

Semantics processing of English sentences using statistical computation based on neurophysiological models

Marcia T. Mitchell

Computer and Information Sciences Department, Saint Peter's University, Jersey City, New Jersey, USA, mmitchell@saintpeters.edu

The examples in this supplementary document will demonstrate how the neuronal networks in the anterior temporal lobe might process sentences. A few examples will present partial semantic of a sentence when the first verb is not the main verb in the sentence. Sentences with a root sentence and subordinate clause will show how the semantic neuronal network might perform parallel processing where the two parts of the sentence execute simultaneously. A few examples will demonstrate how the semantic neuronal network selects prepositional phrases to be included in the semantics.

This research presented four types of semantics that the neuronal network might produce. This supplementary document will present the partial semantic II that was mentioned in the research paper. The sentences used in this research were from the New York Times Corpus (Sandaus, 2008) that have more than twenty words. A few sentences have more than fifty words, which allowed the researcher to find the patterns in the sentence structure.

The Simulation of the Semantic Neuronal Network

A computational model that is represented at the behavioral level of the neuron will show how the information in the semantic neuronal network is represented in convergence and divergence projection of the neuronal network during processing.

EXAMPLE #1

Example #1 will demonstrate the partial semantic II. The sentence has fifty-nine words with four verb and noun phrase patterns.

Once a sentence has been broken down into categories, the MAYA Semantic Technique is applied, which involves the following steps:

The MAYA Semantic Technique begins with grouping related categories. Hence, adjectives are grouped with nouns, and adverbs are grouped with verbs. It is important to note that the prepositional phrases are not included as part of the verb and noun phrase groups. Prepositional phrases are only recognized during semantic interpretation of a sentence. The natural language processing program changes all the words in the sentence to lower case.

Step 1: Determine the categories and identify the phrases in the sentence.

Mr. Rubin, now the chairman of the executive committee at Citigroup, was responding to a question posed to him about whether the 20 percent fee on profits that most private equity firms charge should continue to be taxed at the lower capital gains rate of 15 percent or changed to the top ordinary income tax rate of 35 percent.

Each word in the sentence below is represented as a neuron.
mister rubin, now the chairman of the executive committee at citigroup, was responding to a question posed to him about whether the 20 percent fee on profits that most private equity firms charge should continue to be taxed at the lower capital gains rate of 15 percent or changed to the top ordinary income tax rate of 35 percent.

Each category below is also represented as a neuron.
*noun noun * adverb art noun prep art adject noun prep noun * verb verb prep art noun verb prep pnoun prep prep art adject noun noun prep noun prep adject adject noun noun noun verb verb prep verb verb prep art adjective noun noun noun prep adject noun conjun verb prep art adject adject noun noun noun prep adject noun*

Each category is represented as an extended ASCII character. The sentence is grouped into verb and noun phrases only in order to determine the number of each group.

Sentence = noun verb noun verb noun verb noun verb noun

The categories are grouped into an equation with plus signs as shown in Equation 1.

$$S = n + v + n + v + n + v + n + v + n$$

Equation 1. The equation for the entire semantic neuronal network.

Equation 1 and Figure 1 represent the entire semantic neuronal network with all its verb and noun phrases. Figure 1 represents the conceptual model of the entire sentence depicted as a semantic neuronal network.

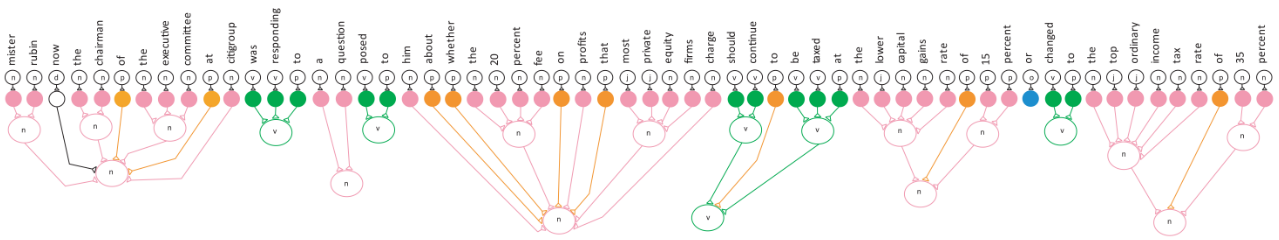

Figure 1. The entire semantic neuronal network for the first sentence.

Step 2: Locate the first verb, "was responding" in the sentence.
Step 3: Determine the subject of the sentence and the words that may come before the first verb in the sentence.

Subject = mister rubin

Step 4: Remove the subject from the sentence.

Equation 2 and Figure 2 represent the semantic neuronal network after the subject was removed.

$$S = v + n + v + n + v + n + v + n$$

Equation 2. The equation for the semantic neuronal network without the subject.

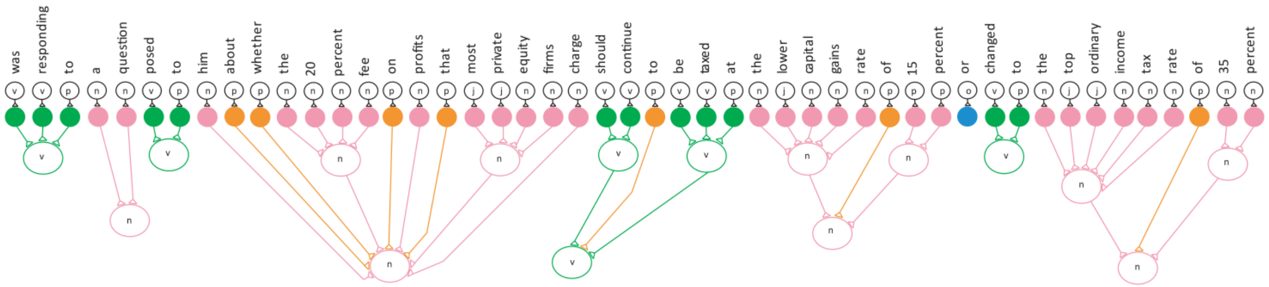

Figure 2. The semantic neuronal network for Equation 2.

The Frequency Technique

Step 5: Neurons summates using nonlinear summation.

The verb and noun phrases in Equation 2 are summed using nonlinear summation. Equation 3 and Figure 3 represent the semantic neuronal network as it depicts the frequency of the verb and noun phrases.

$$S_0 = v^4 + n^4$$

Equation 3. The frequency for both the verb and noun phrase.

Figure 3 represents the conceptual model of the convergence projection of the neuronal network that will be used to derive the frequency for both the verb and noun phrases.

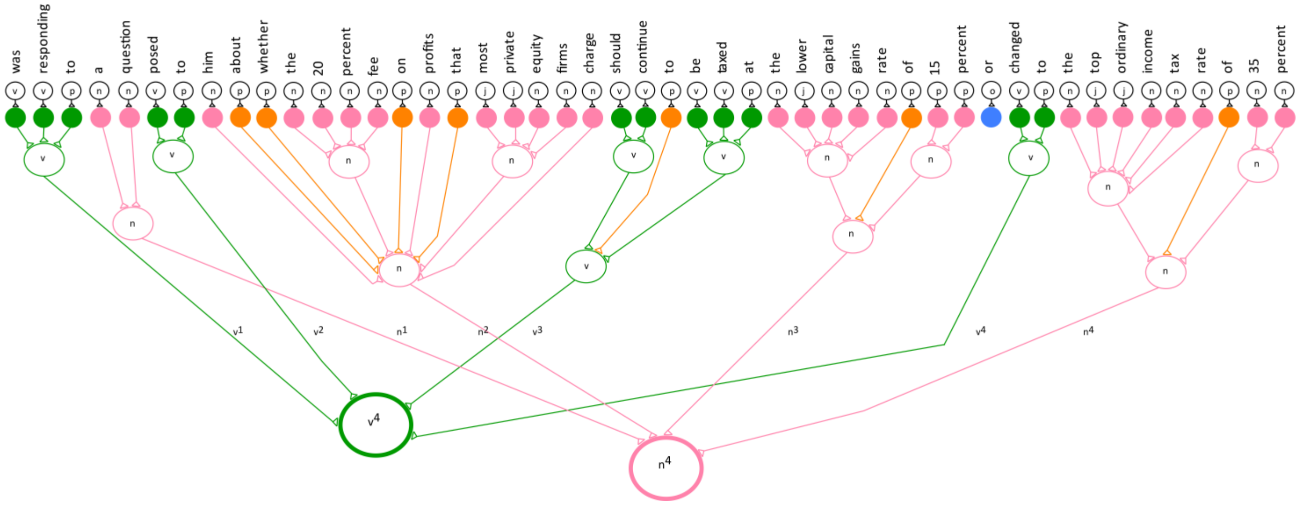

Figure 3. Convergence projection of the neuronal network for the verb and noun phrase.

The Reduction Technique

Step 6: Reduce until the verb is in its lowest term (v^1).

Table 1 shows the verb and noun phrase group for the sentence, where **$S = v^4 + n^4$** points to the last verb and noun phrase group in the sentence. Figure 4 depicts the divergence projection of the neuronal network for the entire sentence.

The reduction	The corresponding words for the verb and noun phrase
$S = v^4 + n^4$	changed to the top ordinary income tax rate of 35 percent
$S_1 = v^3 + n^3$	should continue to be taxed at the lower capital gains rate of 15 percent or
$S_2 = v^2 + n^2$	posed to him about whether the 20 percent fee on profits that most private equity firms charge
$S_3 = v^1 + n^1$	was responding to a question

Table 1. The verb and noun phrase patterns in the sentence.

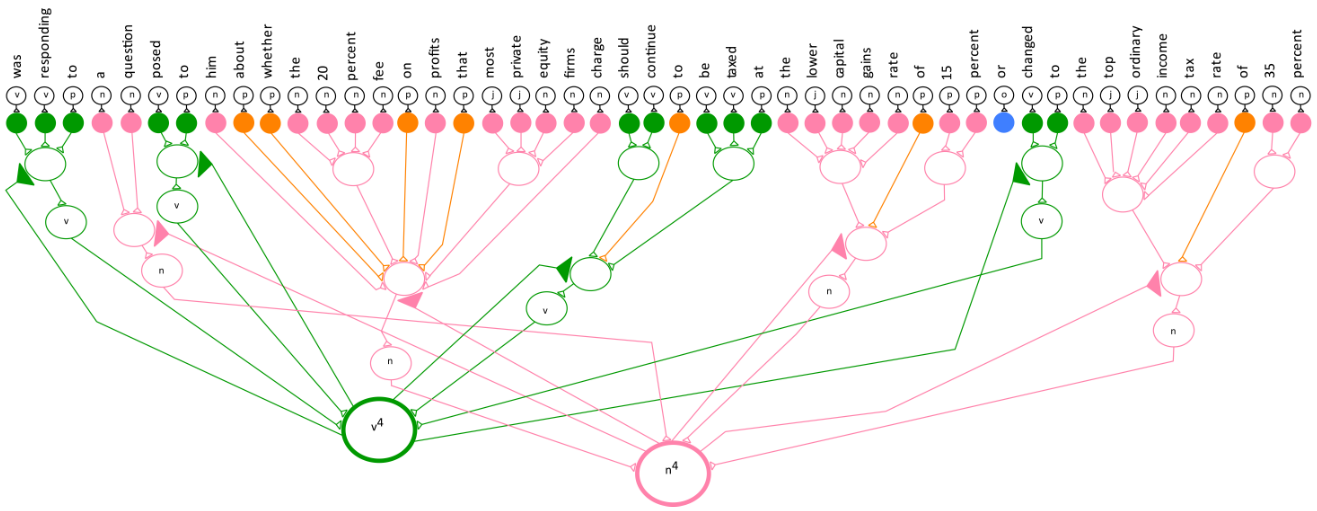

Figure 4. The divergence projection of the semantic neuronal network.

As shown in Table 1 the general equation of the sentence after the subject has been removed:

$$S = v^1 + n^1 + v^2 + n^2 + v^3 + n^3 + v^4 + n^4$$

Equation 4. The general equation.

Where **$v^4 + n^4$** represents the fourth verb and noun phrase group in the sentence.

The general equation of the sentence will be used to determine the full semantic, partial semantic I and partial semantic III.

The Full Semantic

The full semantic of the sentence is:
Full semantic = $V^1 + N^1 + V^2 + N^2 + V^3 + N^3 + V^4 + N^4$

The noun phrase (np) and the prepositional phrase will represent the object for that group**. Figure 4** depicts the entire sentence with its full semantics.

Category	Abbreviation
noun phrase	np
prepositional phase	pp

Example patterns	The object
np + pp + pp	np + pp
pp + np + pp	pp
pp + pp + np	pp

This research have found the following prepositional phrase patterns. These patterns are used as the object within each verb and object group.

Example patterns	The object
$pp_1 + pp_2 + pp_3$	$pp_1 + pp_3$
$pp_1 + pp_2 + pp_3 + pp_4$	$pp_2 + pp_4$
$pp_1 + pp_2 + pp_3 + pp_4$	$pp_1 + pp_4$
$pp_1 + pp_2 + pp_3 + pp_4 + pp_5$	$pp_1 + pp_3 + pp_5$
$pp_1 + pp_2 + pp_3 + pp_4 + pp_5 + pp_6 + pp_7 + pp_8 + pp_9$	$pp_1 + pp_5 + pp_9$

Table 2. Prepositional phrase patterns.

Each verb and noun group in Figure 4 represents the verb and its object. The verb and its object patterns are listed in Table 3. In this sentence all objects are prepositional phrases.

General equation	The specific equation for each verb and noun phrase group
$V^1 + N^1$	Verb + Prepositional Phrase
$V^2 + N^2$	Verb + Prepositional $Phase_1$ + Prepositional $Phase_3$ + Prepositional $Phase_5$
$V^3 + N^3$	Verb + Prepositional $Phase_1$
$V^4 + N^4$	Verb + Prepositional $Phase_1$

Table 3. The verb and its object for the full semantics.

The verb and noun group patterns along with the corresponding words for the sentence are listed in Table 4.

General equation	The corresponding words for the equation
$V^1 + N^1$	was responding to a question
$V^2 + N^2$	posed to him whether the 20 percent fee that most private equity firms charge
$V^3 + N^3$	should continue to be taxed at 15 percent or
$V^4 + N^4$	changed to 35 percent

Table 4. The actual phrases that make up the full semantcis.

The full semantic produces an almost exact reproduction of the original sentence. Table 4a shows the second verb and noun phrase group in the sentence where the two prepositional phrases are excluded from the semantic.

Original text	$S_2 = v^2 + n^2$	posed to him about whether the 20 percent fee on profits that most private equity firms charge
Reduced text from the full semantic	$V^2 + N^2$	posed to him whether the 20 percent fee that most private equity firms charge

Table 4a. The exclusion of two prepositional phrases.

Table 4b shows the two prepositional phrases that were not included as part of the full semantic.

	Prepositional Phrase$_1$	Prepositional Phrase$_2$	Prepositional Phrase$_3$	Prepositional Phrase$_4$	Prepositional Phrase$_5$
Original text	to him	about	whether the 20 percent fee	on profits	that most private equity firms charge
Reduced text	to him		whether the 20 percent fee		that most private equity firms charge

Table 4b. The Prepositional Phrase$_2$ and Prepositional Phrase$_4$ are not included in the full semantic.

The full semantics, shown below, produces an almost exact reproduction of the original sentence but leaves out some details.

mister rubin, now the chairman of the executive committee at citigroup was responding to a question posed to him whether the 20 percent fee that most private equity firms charge should continue to be taxed at the lower capital gains rate or changed to the top ordinary income tax rate.

Notice that p_2 and p_4 were not included in the object for the second verb and noun group pattern ($V^2 + N^2$) because the neuronal network produce a slightly smaller version of the sentence. The full semantic leaves out details that are not needed for comprehension.

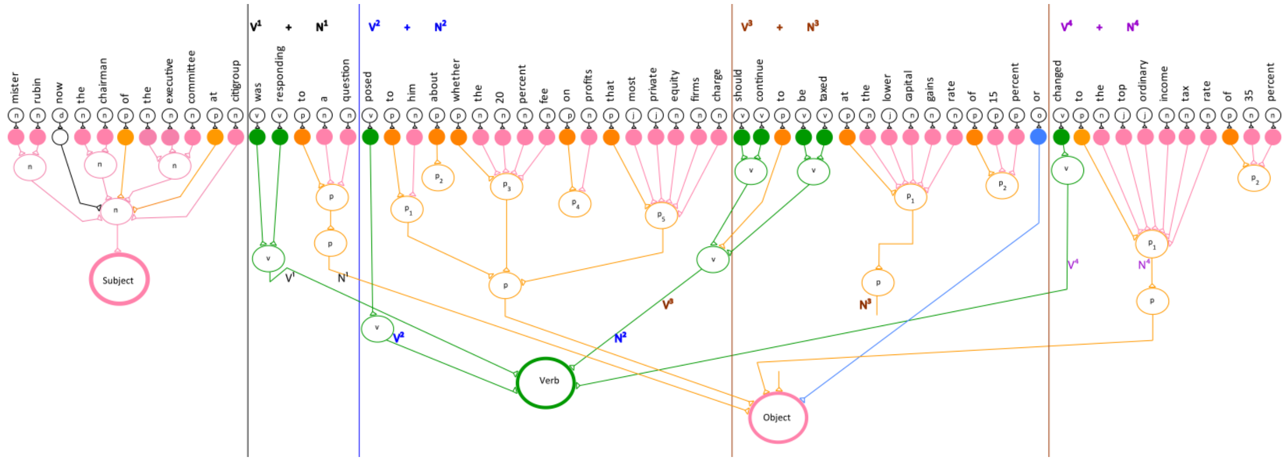

Figure 5. The full semantic for the first sentence.

The Partial Semantic I

The general equation for the partial semantic I:

$$\text{Partial Semantic I} = \text{Verb}^1 + \text{noun}_1^1 + \text{noun}_y^1 + \text{Verb}^2 + \text{noun}_1^2 + \text{noun}_y^2 + \text{Verb}^3 + \text{noun}_1^3 + \text{noun}_y^3 + \cdots \text{Verb}^m + \text{noun}_1^m + \text{noun}_y^m$$

The partial semantic for the sentence is:

$$\text{Partial Semantic I} = \text{Verb}^1 + \text{noun}_y^1 + \text{Verb}^2 + \text{noun}_1^2 + \text{noun}_y^2 + \text{Verb}^3 + \text{noun}_1^3 + \text{noun}_y^3 + \text{Verb}^4 + \text{noun}_1^4 + \text{noun}_y^4$$

Table 5 shows the equations for each verb and noun phrase group within the sentence. Figure 6 shows the partial semantic I for the sentence.

The specific equation	The corresponding words for the equation
$\text{Verb}^1 + noun_y^1$	was responding to a question
$\text{Verb}^2 + noun_1^2 + noun_y^2$	posed to him about whether most private equity firms charge
$\text{Verb}^3 + noun_1^3 + noun_y^3$	should continue to be taxed at lower capital gains rate 15 percent or
$\text{Verb}^4 + noun_1^4 + noun_y^4$	changed to top ordinary income tax rate 35 percent

Table 5. The phrases associated with the partial semantic I.

The partial semantic I, shown below, produces a shorter version of the full semantics:

mister rubin, now the chairman of the executive committee at citigroup was responding to a question posed to him about whether private equity firms charge should continue to be taxed at the lower capital gains rate of 15 percent or changed to income tax rate of 35 percent.

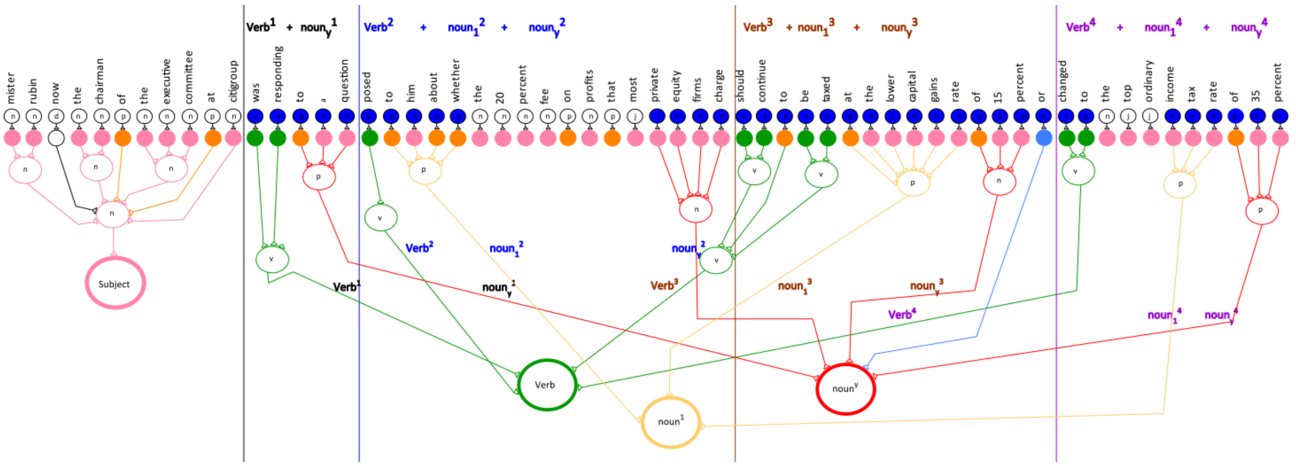

Figure 6. The partial semantic I for the first sentence.

The Partial Semantic II

The partial semantic II produces a slightly smaller version of the sentence than the partial semantic I. Partial semantic II includes the verb and the last noun phrase within each verb and noun phrase group. The processing for the partial semantic II is a lateral excitation.

Figure 7 shows verb and noun phrases that are included in the partial semantics II.

The general equation for the partial semantic II.

$$\text{Partial Semantic II} = \text{Verb}^1 + noun_y^1 + \text{Verb}^2 + noun_y^2 + \text{Verb}^3 + noun_y^3 + \ldots\ \text{Verb}^m + noun_y^m$$

The partial semantic II for the sentence is:

$$\text{Partial Semantic II} = \text{Verb}^1 + noun_y^1 + \text{Verb}^2 + noun_y^2 + \text{Verb}^3 + noun_y^3 + \text{Verb}^4 + noun_y^4$$

Table 6 shows the equations for each verb and noun phrase group within the sentence. Figure 7 shows the partial semantic II for the sentence.

The specific equation	The corresponding words for the equation
$\text{Verb}^1 + noun_y^1$	was responding to a question
$\text{Verb}^2 + noun_y^2$	posed most private equity firms charge
$\text{Verb}^3 + noun_y^3$	should continue to be taxed at 15 percent or
$\text{Verb}^4 + noun_y^4$	changed to 35 percent

Table 6. The phrases associated with the partial semantic II.

The partial semantic I, shown below, produces a general overview of the sentence and it can be computed for some sentences.

mister rubin now the chairman of the executive committee at citigroup was responding to question posed most private equity firms charge should continue to be taxed at 15 percent or changed to 35 percent.

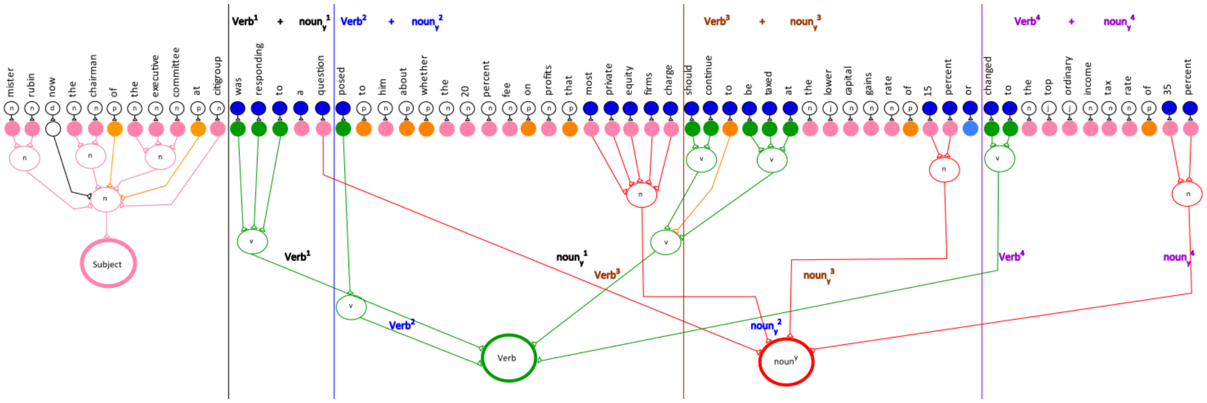

Figure 7. Partial semantic II for the first sentence.

The Partial Semantic III

The partial semantic III produces a brief meaning of the sentence which includes the main verb and its object. Figure 8 displays the subject, verb and its object that comprise the partial semantic III for the sentence.

Subject	Verb	Object/Complement
mister rubin now the chairman of the executive committee at citigroup	was responding	to a question

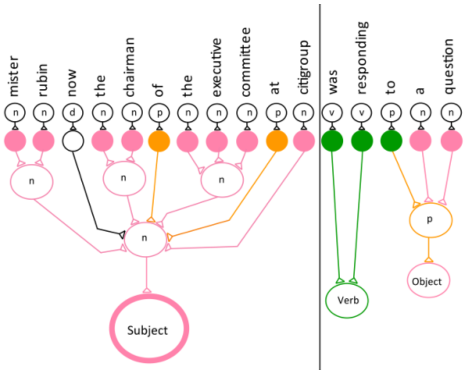

Figure 8. The partial semantics III for the first sentence.

EXAMPLE #2

Example #2 will demonstrate processing a sentence with a subordinate clause. The sentence has forty-five words with six verb and noun phrase patterns.

Step 1: Determine the categories and identify the phrases in the sentence.

As part of the agreement, State Farm was insisting that Jim Hood, the attorney general of Mississippi, drop a criminal investigation into the company's handling of claims and call off the grand jury, which would decide whether there was probable cause to return an indictment.

Each word in the sentence below is represented as a neuron.
state farm was insisting that jim hood, the attorney general of mississippi, drop a criminal investigation into the company handling of claims and call off the grand jury, which would decide whether there was probable cause to return an indictment.

Each category below is also represented as a neuron.
*noun noun verb verb art noun noun * art noun noun prep noun * verb art adject noun prep art noun noun prep noun conjun verb adverb art adject noun * art verb verb prep pnoun verb adject noun prep verb art noun*

Each category is represented as an extended ASCII character.

Sentence = noun verb noun verb noun verb noun verb noun verb noun verb noun

The categories are grouped into an equation with plus signs as shown in Equation 5.

$$S = n + v + n + v + n + v + n + v + n + v + n + v + n$$

Equation 5. The equation for the entire semantic neuronal network.

Equation 5 and Figure 9 represent the entire semantic neuronal network with all its verb and noun phrases. Figure 9 represents the conceptual model of the entire sentence depicted as a semantic neuronal network.

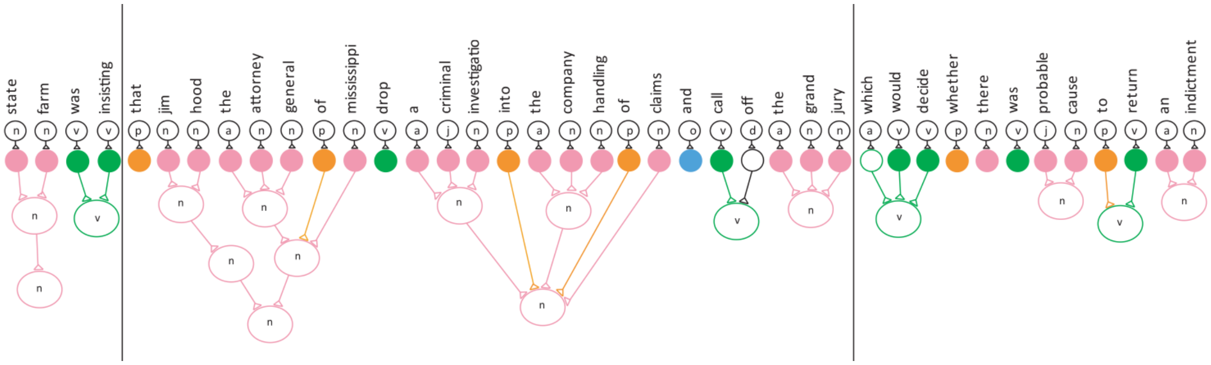

Figure 9. The entire semantic neuronal network.

Step 2: Locate the first verb "was insisting" in the root sentence.
Locate the first verb "drop" in the subordinate clause.

Step 3: Determine the subject of the sentence and the words that may come before the first verb in the sentence.

The sentence has compound subjects:

$\text{Subject}_{\text{root}}$	state farm
$\text{Subject}_{\text{subordinate clause}}$	jim hood

Step 4: Remove the subject from the sentence.

Equation 6 and Figure 10 represent the semantic neuronal network after the subject was removed.

$$S = v + n + v + n + v + n + v + n + v + n + v + n$$

Equation 6. The equation for the semantic neuronal network without the subject.

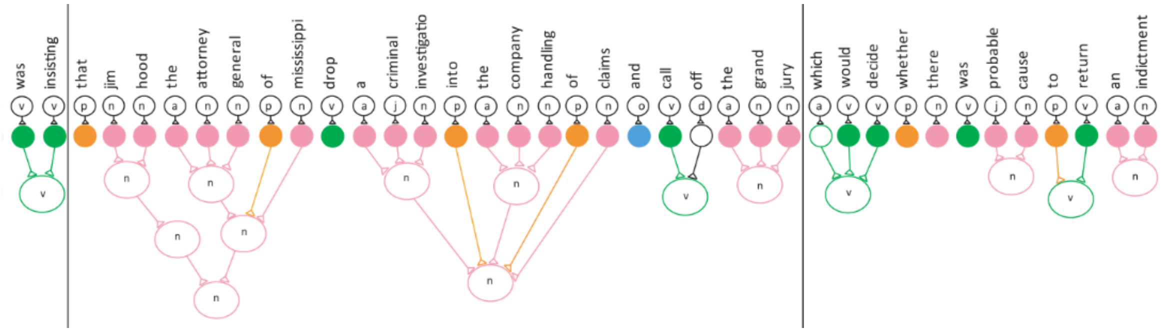

Figure 10. The semantic neuronal network for Equation 6.

The Frequency Technique

Step 5: Neurons summates using nonlinear summation.

The verb and noun phrases in Equation 6 are summed using nonlinear summation. Equations 7 and 7a and Figure 11 represent the semantic neuronal network as it depicts the frequency of the verb and noun phrases.

Root sentence

$$S_0 = v^3 + n^3$$

Equation 7. The frequency for both the verb and noun phrase.

Subordinate clause

$$S_1 = v^2 + n^2$$

Equation 7a. The frequency for both the verb and noun phrase.

Figure 11 represents the conceptual model of the convergence projection of the neuronal network that will be used to derive the frequency for both the verb and noun phrases.

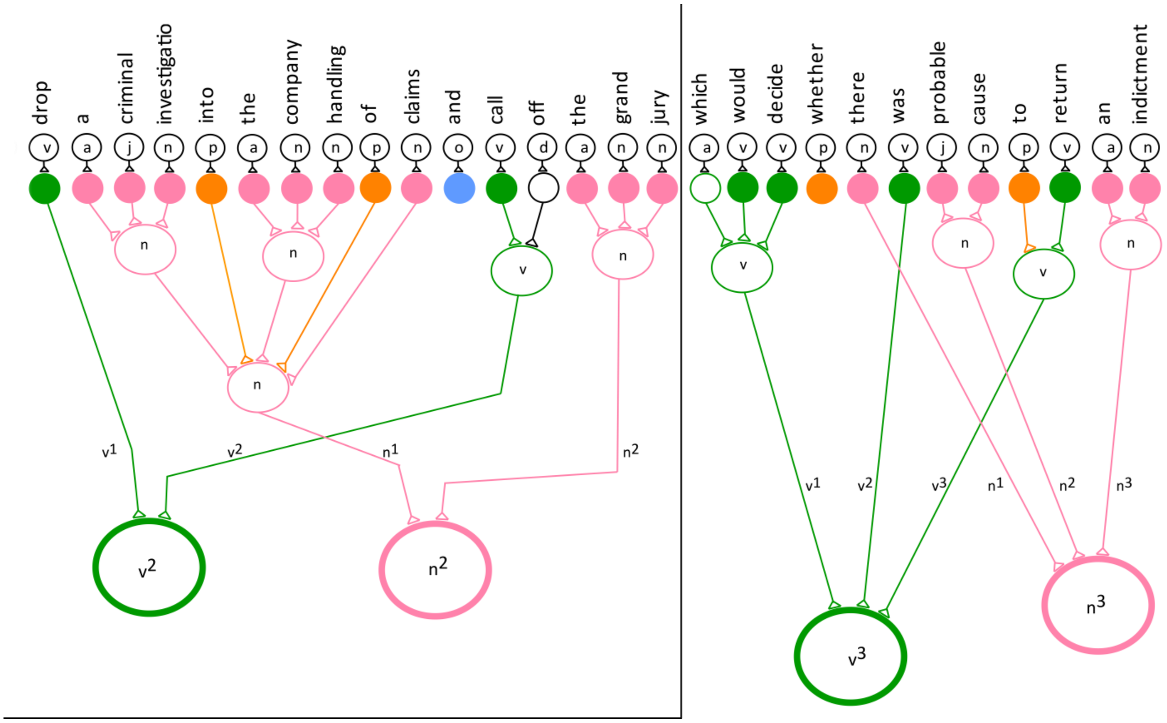

Figure 11. The convergence projection of the semantic neuronal network.

The Reduction Technique

Step 6: Reduce until the verb is in its lowest term (v^1).

Table 7 shows the verb and noun phrase group for the sentence, where $\mathbf{S = v^3 + n^3}$ points to the last verb and noun phrase group in the sentence. Figure 12 depicts the divergence projection of the neuronal network for the entire sentence.

Root Sentence	
**The reduction**	**The corresponding words for the verb and noun phrase**
$S_0 = v^3 + n^3$	to return an indictment
$S_1 = v^2 + n^2$	was probable cause
$S_2 = v^1 + n^1$	which would decide whether there

Table 7. The verb and noun phrase patterns in the root sentence.

The verb and noun group patterns along with the corresponding words for the sentence are listed in Table 7a.

Subordinate clause	
**The reduction**	**The corresponding words for the verb and noun phrase**
$S_0 = v^2 + n^2$	call off the grand jury
$S_1 = v^1 + n^1$	drop a criminal investigation into the company handling of claims and

Table 7a. The verb and noun phrase patterns in the subordinate clause.

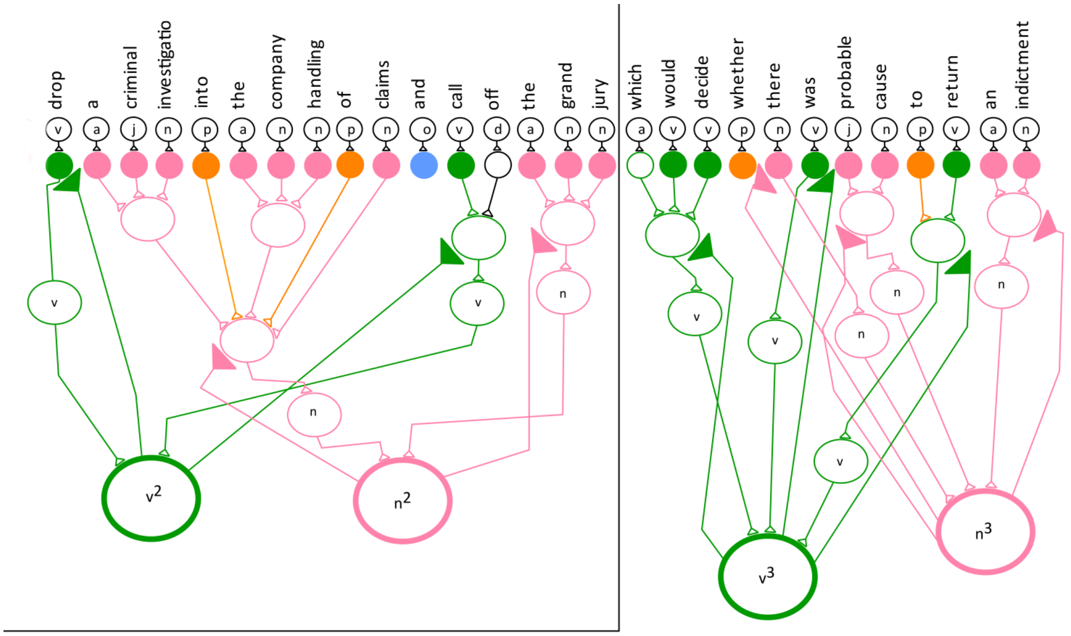

Figure 12. The divergence projection of the semantic neuronal network.

$S = v^1 + n^1 + v^2 + n^2 + v^3 + n^3 + v^4 + n^4 + v^5 + n^5 + v^6 + n^6$

Equation 8. The general equation.

The Full Semantics

The full semantics of the sentence is:
Full semantics = $V^1 + N^1 + V^2 + N^2 + V^3 + N^3 + V^4 + N^4 + V^5 + N^5 + V^6 + N^6$

The verb and object patterns for this sentence is listed in Table 8.

General equation	The specific equations for the each verb and noun group
$V^1 + N^1$	Verb + Prepositional phrase
$V^2 + N^2$	Verb + Noun Phrase + Prepositional Phase
$V^3 + N^3$	Verb + Noun Phrase
$V^4 + N^4$	Verb + Prepositional Phase
$V^5 + N^5$	Verb + Noun Phrase
$V^6 + N^6$	Verb + Noun Phrase

Table 8. The verb and its object for the full semantics.

The verb and noun phrase pattern for the sentence along with the words are listed in Table 9. Figure 13 shows the full semantic for the sentence.

General equation	The corresponding words in the equation
$V^1 + N^1$	was insisting that jim hood
$V^2 + N^2$	drop a criminal investigation into the company handling
$V^3 + N^3$	call off the grand jury
$V^4 + N^4$	which would decide whether there
$V^5 + N^5$	was probable cause
$V^6 + N^6$	to return an indictment

Table 9. The actual phrases that make up the full semantcis.

The full semantics:

state farm was insisting that jim hood drop a criminal investigation into the company handling call off the grand jury, which would decide whether there was probable cause to return an indictment.

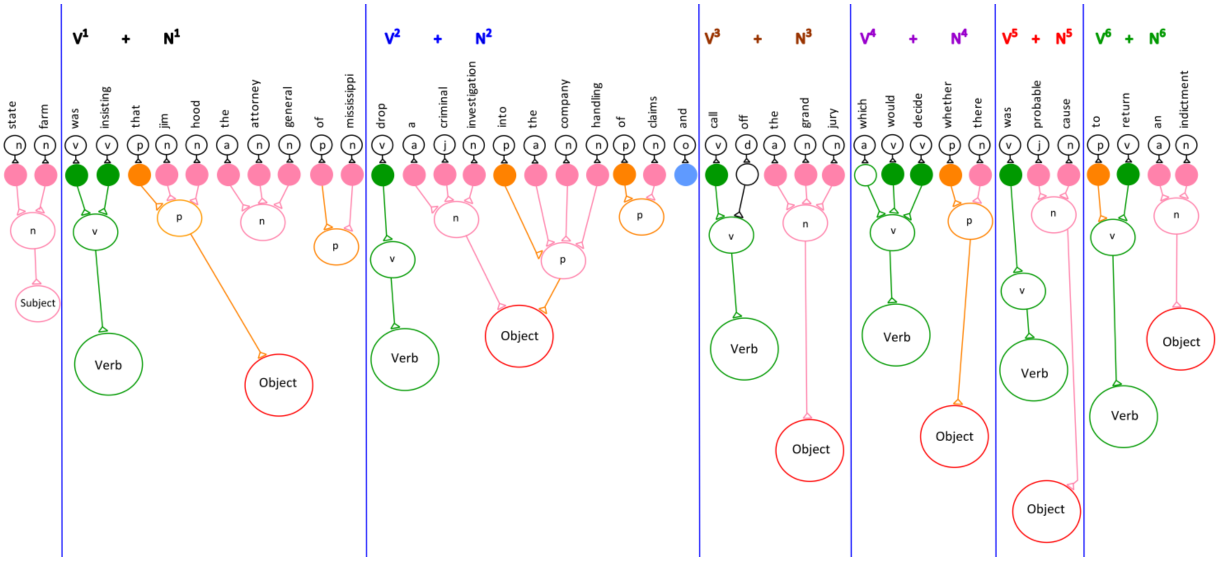

Figure 13. The full semantic for the second sentence.

The Partial Semantic I

The Partial Semantic I for the sentence is:

Partial Semantic I = $\text{Verb}^1 + noun_1^1 + noun_y^1 + \text{Verb}^2 + noun_y^2 + \text{Verb}^3 + noun_y^3 + \text{Verb}^4 + noun_y^4 + \text{Verb}^5 + noun_y^5$

Table 10 and 10a show the equations for each verb and noun phrase group within the sentence. Figure 14 shows the partial semantic I for the sentence.

Root Sentence	
**The specific equation**	**The corresponding words for the equation**
$\text{Verb}^3 + noun_y^3$	would decide whether there
$\text{Verb}^4 + noun_y^4$	was probable cause
$\text{Verb}^5 + noun_y^5$	to return an indictment

Table 10. The phrases associated with the partial semantic I for the root sentence.

Subordinate Clause	
**The specific equation**	**The corresponding words for the equation**
$\text{Verb}^1 + noun_1^1 + noun_y^1$	drop criminal investigation of claims
$\text{Verb}^2 + noun_y^2$	call off grand jury

Table 10a. The phrases associated with the partial semantic I for the subordinate clause.

Partial semantic I for the sentence:

state farm was insisting that jim hood the attorney general of mississippi drop criminal investigation of claims call off grand jury would decide whether there was cause to return indictment

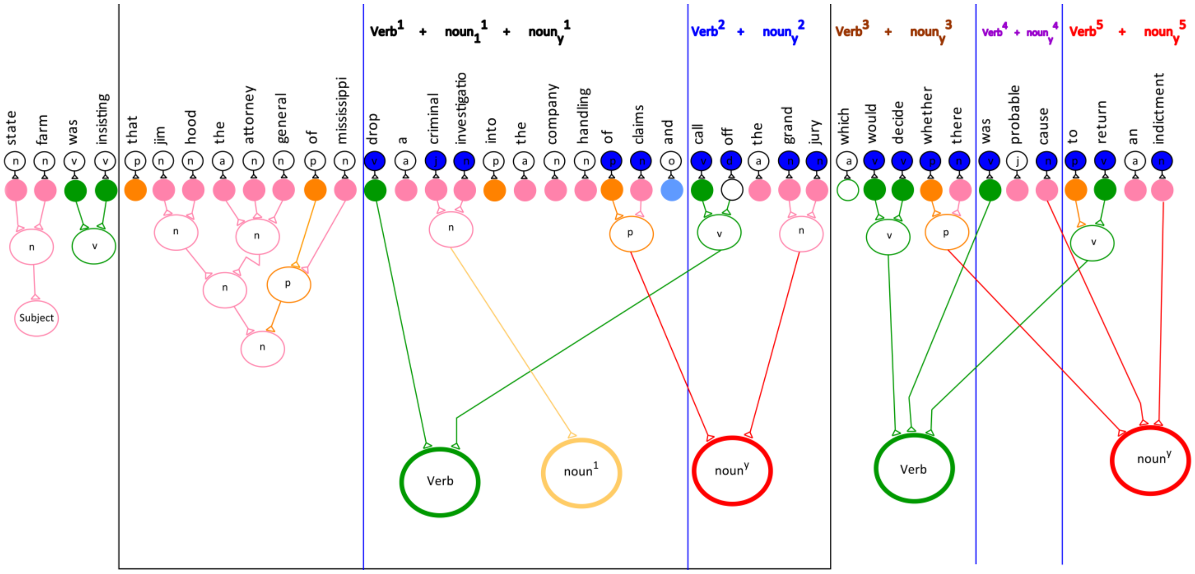

Figure 14. Partial semantics I for the second sentence.

The Partial Semantic III

Figure 15 shows the partial semantic III for the sentence.

Subject	Verb	Object/Complement
state farm	was insisting	
jim hood the attorney general of mississippi	drop	a criminal investigation into the company handling

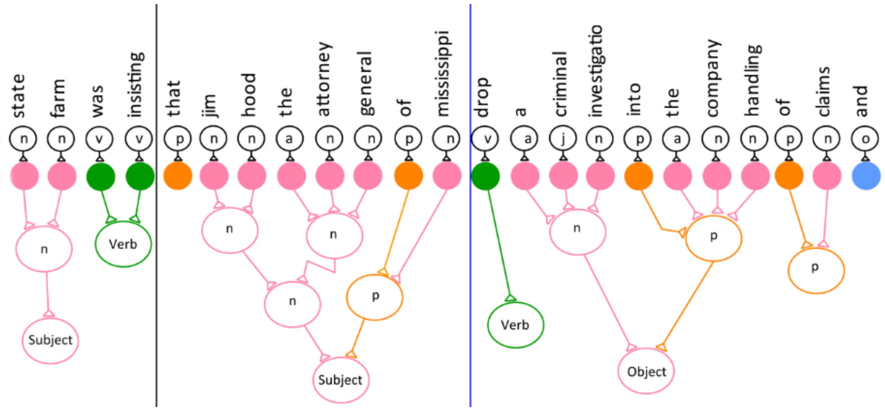

Figure 15. Partial semantics III for the second sentence.

EXAMPLE #3
Example #3 will demonstrate the subordinate clause. The sentence has twenty-six words with three verb and noun phrase patterns.

Step 1: Determine the categories and identify the phrases in the sentence.

Harriet E. Miers, the Supreme Court nominee, disclosed on Tuesday a 1989 survey in which she supported banning abortion except to protect the life of the pregnant woman.

Each word in the sentence below is represented as a neuron.
harriet miers the supreme court nominee disclosed on tuesday 1989 survey in which she supported banning abortion except to protect the life of the pregnant woman.

Each category below is also represented as a neuron.
noun noun art adject noun noun verb prep name adject noun prep art pnoun verb verb noun prep prep verb art noun prep art adject noun

Each category is represented as an extended ASCII character. The sentence is grouped into verb and noun phrases only in order to determine the number of each group.

Sentence = noun verb noun verb noun verb noun

The categories are grouped into an equation with plus signs as shown in Equation 9.

$$S = n + v + n + v + n + v + n$$

Equation 9. The equation for the entire semantic neuronal network.

Equation 9 and Figure 16 represent the entire semantic neuronal network with all its verb and noun phrases. Figure 16 represents the conceptual model of the entire sentence depicted as a semantic neuronal network.

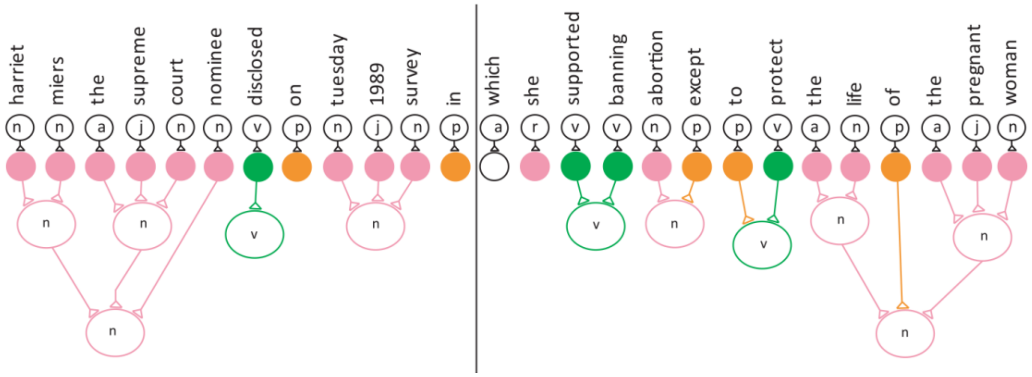

Figure 16. The entire semantic neuronal network.

Step 2: Locate the first verb, “disclosed” in the sentence.

Step 3: Determine the subject of the sentence and the words that may come before the first verb in the sentence.

Subject = harriet miers

Step 4: Remove the subject from the sentence.

Equation 10 and Figure 17 represent the semantic neuronal network after the subject was removed.

$$S = v + n + v + n + v + n$$

Equation 10. The equation for the semantic neuronal network without the subject.

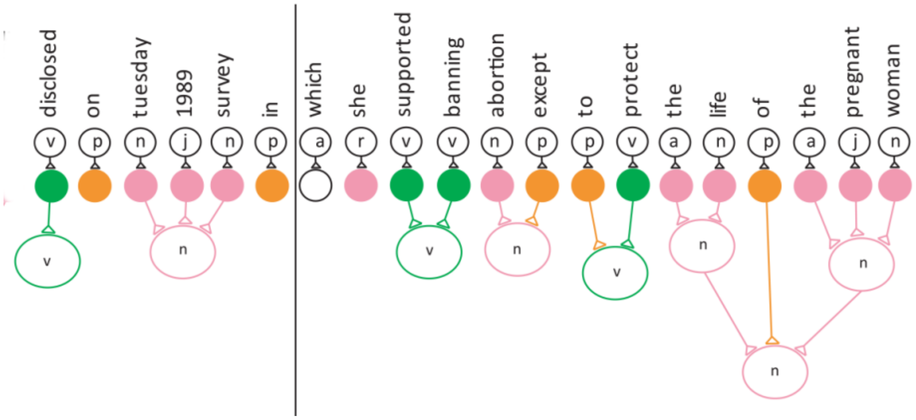

Figure 17. The semantic neuronal network for Equation 10.

The Frequency Technique

Step 5: Neurons summates using nonlinear summation.

The verb and noun phrases in Equation 10 are summed using nonlinear summation. Equation 11 and Figure 18 represent the semantic neuronal network as it depicts the frequency of the verb and noun phrases.

$$S_0 = v^2 + n^2$$

Equation 11. The frequency for both the verb and noun phrase.

Figure 18 represents the conceptual model of the convergence projection of the neuronal network that will be used to derive the frequency for both the verb and noun phrases.

The Reduction Technique

Step 6: Reduce until the verb is in its lowest term (v^1).

Tables 11 and 11a show the verb and noun phrase group for the sentence.
Figure 19 shows the reduction technique for the subordinate clause.

Root Sentence	
**The reduction**	**The corresponding words for the verb and noun phrase**
$S_0 = v^1 + n^1$	disclosed on tuesday 1989 survey in

Table 11. The verb and noun phrase patterns in the root sentence.

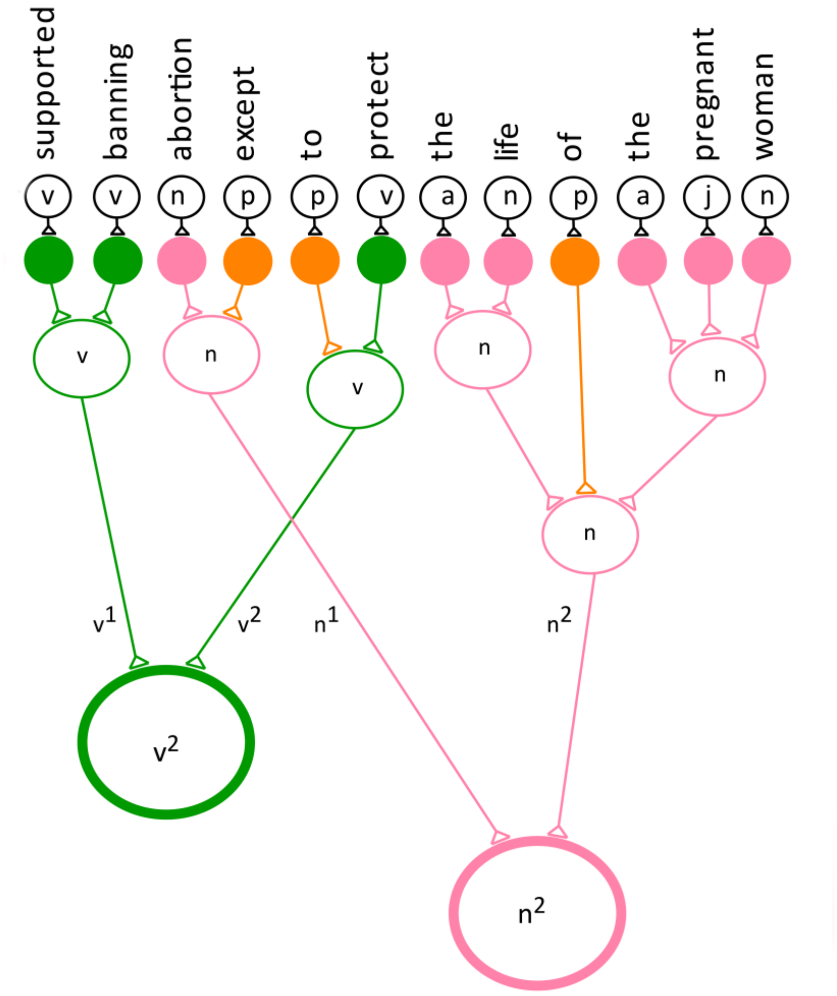

Figure 18. The convergence projection of the semantic neuronal network.

Subordinate Clause	
**The Reduction**	**The corresponding words for the verb and noun phrase**
$S_0 = v^2 + n^2$	to protect the life of the pregnant woman
$S_1 = v^1 + n^1$	supported banning abortion except

Table 11a. The verb and noun phrase patterns in the subordinate claue.

$$S = v^1 + n^1 + v^2 + n^2 + v^3 + n^3$$

Equation 12. The general equation.

The Full Semantics

The full semantics of the sentence is:

Full semantics = $V^1 + N^1 + V^2 + N^2$

The verb and object patterns for this sentence is listed in Table 12.

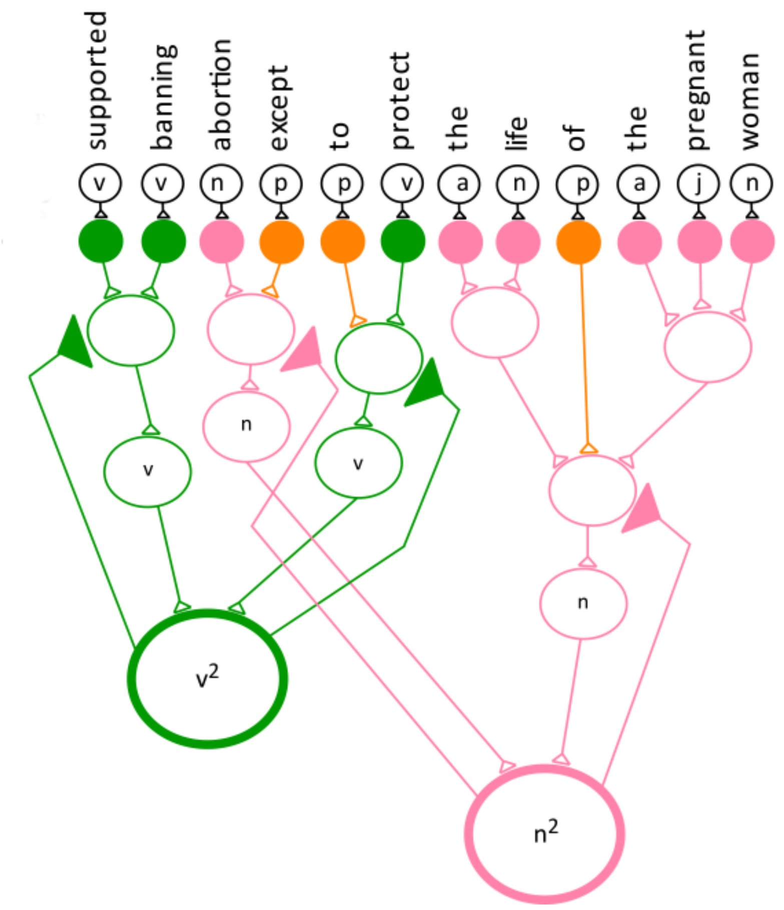

Figure 19. The divergence projection of the semantic neuronal network.

General equation	The specific equation for each verb and noun phrase group
$V^1 + N^1$	Verb + Noun Phrase
$V^2 + N^2$	Verb + Noun Phrase + Prepositional Phase

Table 12. The verb and its object for the full semantics.

The verb and noun phrase pattern for the sentence along with the words are listed in Table 13. Figure 20 shows the full semantic for the sentence.

General equation	The specific equation for each verb and noun phrase group
$V^1 + N^1$	supported banning abortion
$V^2 + N^2$	to protect the life of the pregnant woman

Table 13. The actual phrases that make up the full semantic.

The full semantics for the sentence is:

harriet miers the supreme court nominee disclosed on tuesday 1989 survey she supported banning abortion except to protect the life of the pregnant woman.

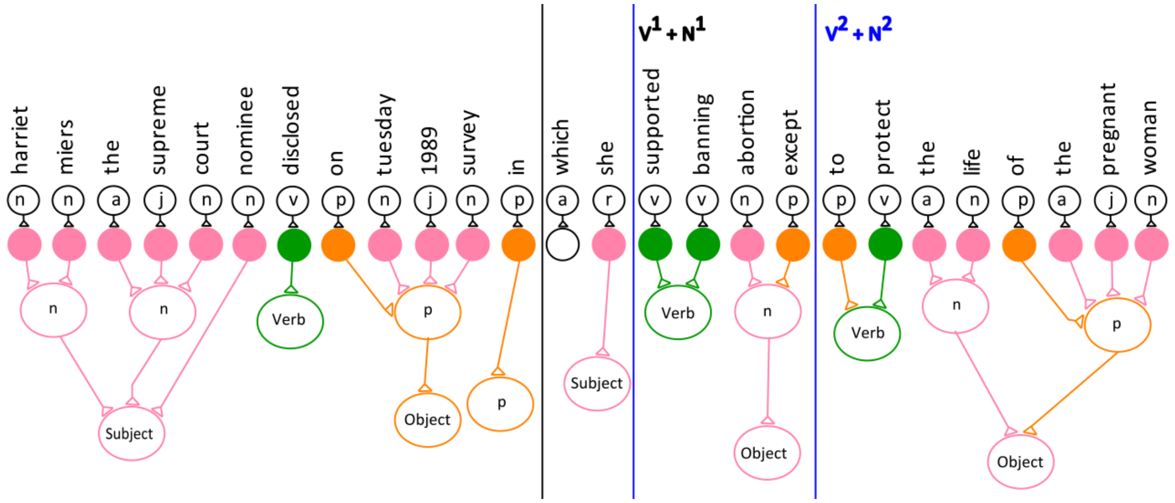

Figure 20. Full semantics for the third sentence.

The Partial Semantic I

The Partial Semantic I for the sentence is:

Partial Semantic I = $\text{Verb}^1 + noun_1^1 + noun_y^1 + \text{Verb}^2 + noun_1^2 + noun_y^2$

Table 14 shows the equations for each verb and noun phrase group within the sentence.

Figure 21 shows the partial semantic I for the sentence.

The specific equation	The corresponding words for the equation
$\text{Verb}^1 + noun_1^1 + noun_y^1$	supported banning abortion except
$\text{Verb}^2 + noun_1^2 + noun_y^2$	to protect life pregnant woman

Table 14. The phrases associated with the partial semantic I.

The partial semantic I for the sentence is:

harriet miers the supreme court nominee disclosed tuesday 1989 survey supported banning abortion except to protect life pregnant woman.

The Partial Semantic II

The Partial Semantic II for the sentence is:

Partial Semantic II = $\text{Verb}^1 + noun_y^1 + \text{Verb}^2 + noun_y^2$

Table 15 shows the equations for each verb and noun phrase group within the sentence. Figure 22 shows the partial semantic II for the sentence.

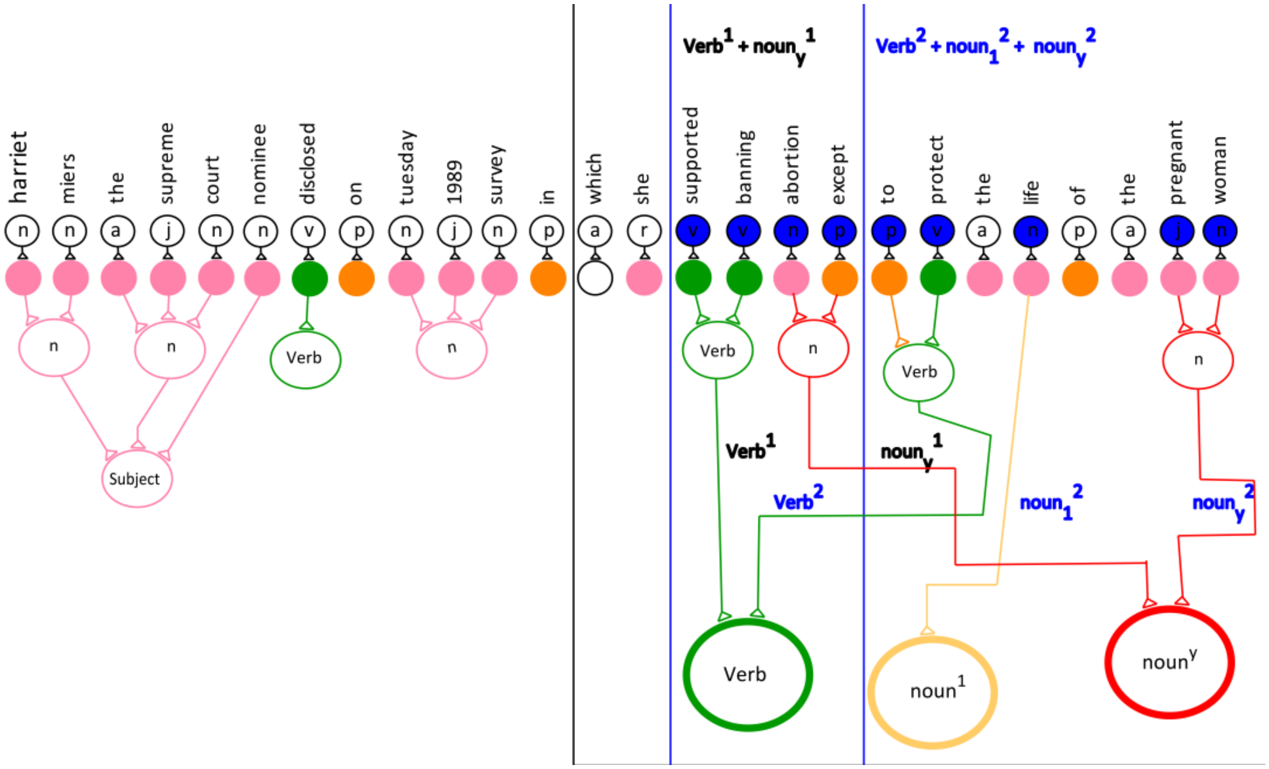

Figure 21. The partial semantics I for the third sentence.

The specific equation	The corresponding words for the equation
$\text{Verb}^1 + noun_y^1$	supported banning abortion except
$\text{Verb}^2 + noun_y^2$	to protect pregnant woman

Table 15. The phrases associated with the partial semantic II.

The partial semantic II for the sentence is:

harriet miers the supreme court nominee disclosed tuesday 1989 survey supported banning abortion except to protect pregnant woman.

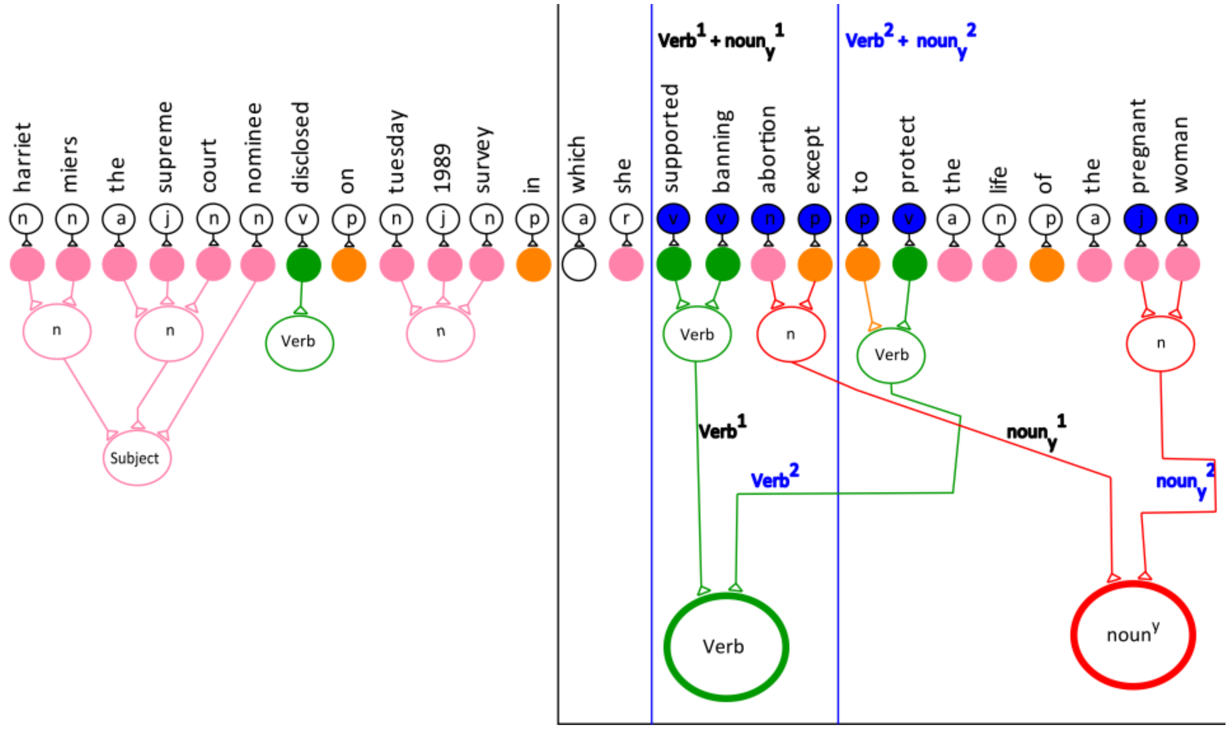

Figure 22. The partial semantic II for the third sentence.

The Partial Semantic III

Figure 23 shows the partial semantic III for the sentence.

Subject	Verb	Object/Complement
harriet miers the supreme court nominee	disclosed	on tuesday 1989 survey
she	supported banning	abortion

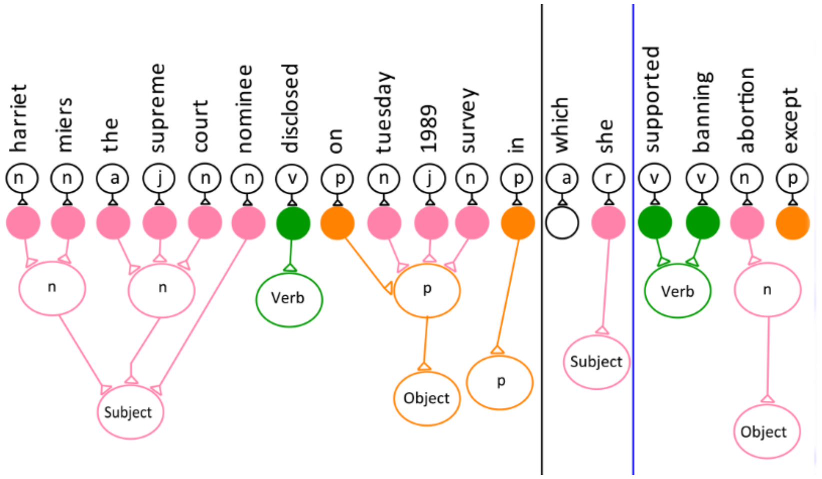

Figure 23. The partial semantic III for the third sentence.

EXAMPLE #4
Example #4 will demonstrate the subordinate clause. The sentence thirty-one words with three verb and noun phrase patterns.

Step 1: Determine the categories and identify the phrases in the sentence.

The Los Angeles city utility, which serves 3.8 million people in Los Angeles, has been offering customers cash rebates of as much as $10,000 a home to convert to solar energy.

Each word in the sentence below is represented as a neuron.
the los angeles city utility, which serves 3.8 million people in los angeles, has been offering customers cash rebates of as much as 10000 a home to convert to solar energy.

Each category below is also represented as a neuron.
*art pnoun pnoun noun noun * art verb adject adject noun prep adject noun * verb verb verb noun noun noun prep adverb adject prep adject art noun prep verb prep adject noun*

Each category is represented as an extended ASCII character.

Sentence = noun verb noun verb noun verb noun verb noun

The categories are grouped into an equation with plus signs as shown in Equation 13.

S = n + v + n + v + n + v + n

Equation 13. The equation for the entire semantic neuronal network.

Equation 13 and Figure 24 represent the entire semantic neuronal network with all its verb and noun phrases. Figure 24 represents the conceptual model of the entire sentence depicted as a semantic neuronal network.

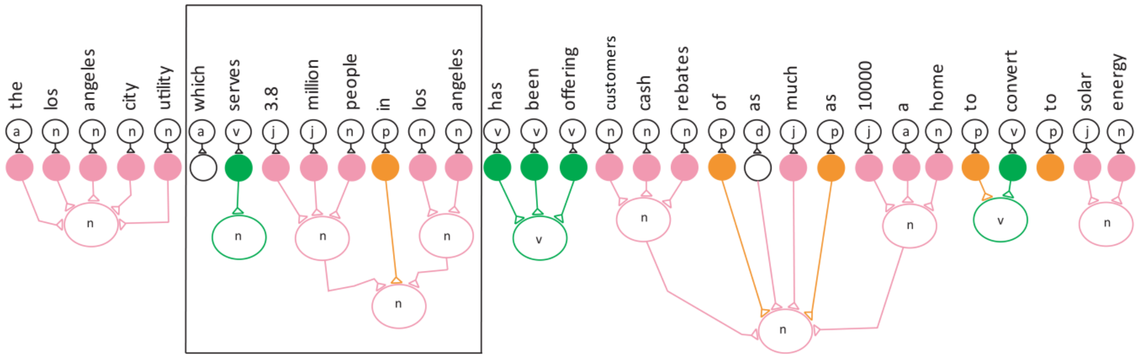

Figure 24. The entire semantic neuronal network.

Step 2: Locate the first verb, "has been offering" in the sentence
Step 3: Determine the subject of the sentence and the words that may come before the first verb in the sentence.

Subject = los angeles city utility

Step 4: Remove the subject from the sentence.

Equation 14 and Figure 25 represent the semantic neuronal network after the subject was removed.

$$S = v + n + v + n + v + n$$

Equation 14. The equation for the semantic neuronal network without the subject.

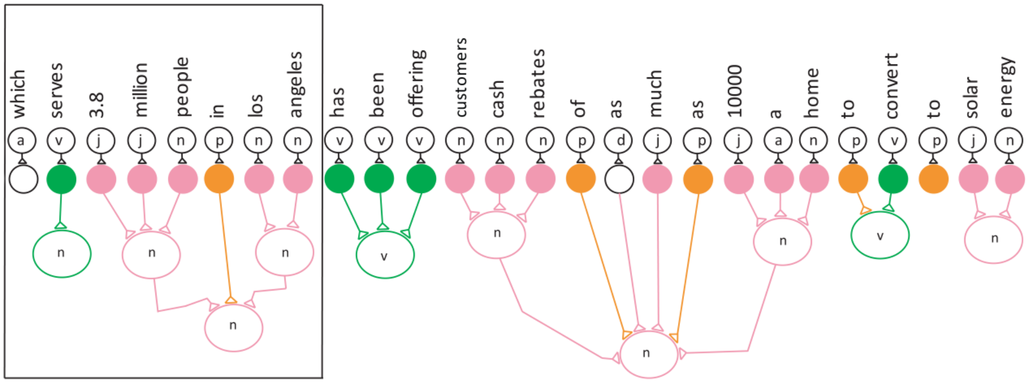

Figure 25. The semantic neuronal network for Equation 14.

The Frequency Technique

Step 5: Neurons summates using nonlinear summation.

The verb and noun phrases in Equation 14 are summed using nonlinear summation. Equations 15 and 15a and Figure 26 represent the semantic neuronal network as it depicts the frequency of the verb and noun phrases.

Subordinate Clause

$$S_0 = v^1 + n^1$$

Equation 15. The frequency for both the verb and noun phrase.

Root Sentence

$$S_0 = v^2 + n^2$$

Equation 15a. The frequency for both the verb and noun phrase.

Figure 26 represents the conceptual model of the convergence projection of the neuronal network that will be used to derive the frequency for both the verb and noun phrases.

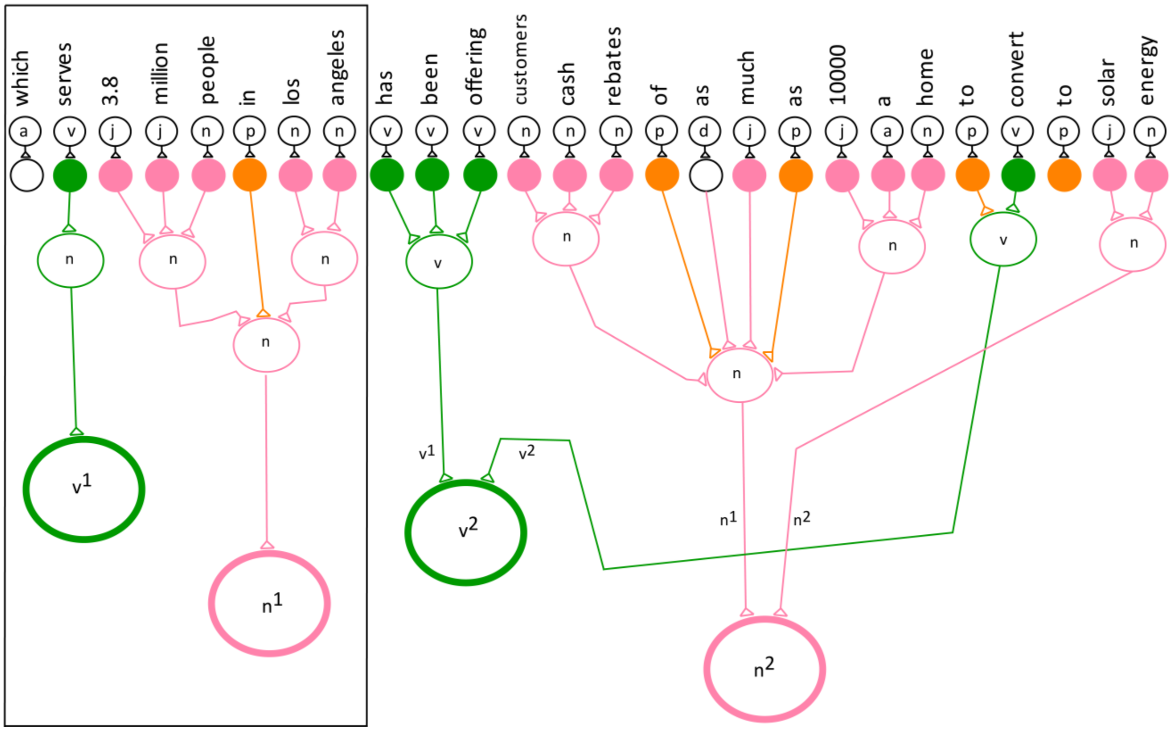

Figure 26. The convergence projection of the semantic neuronal network.

The Reduction Technique

Step 6: Reduce until the verb is in its lowest term (v^1).

Tables 16 and 16a show the verb and noun phrase group for the sentence.

Figure 27 shows the reduction technique for both the root sentence and the subordinate clause.

**Root Sentence**	
**The reduction**	**The corresponding words for the verb and noun phrase**
$S_0 = v^2 + n^2$	customers cash rebates of as much as 10000 a home
$S_1 = v^1 + n^1$	to convert to solar energy

Table 16. The reduction technique for the root sentence.

**Subordinate Clause**	
**The reduction**	**The corresponding words for the verb and noun phrase**
$S_0 = v^1 + n^1$	serves 3.8 million people in los angeles

Table 16a. The reduction technique for the subordinate clause.

$$S = v^1 + n^1 + v^2 + n^2 + v^3 + n^3$$

Equation 16. The general equation.

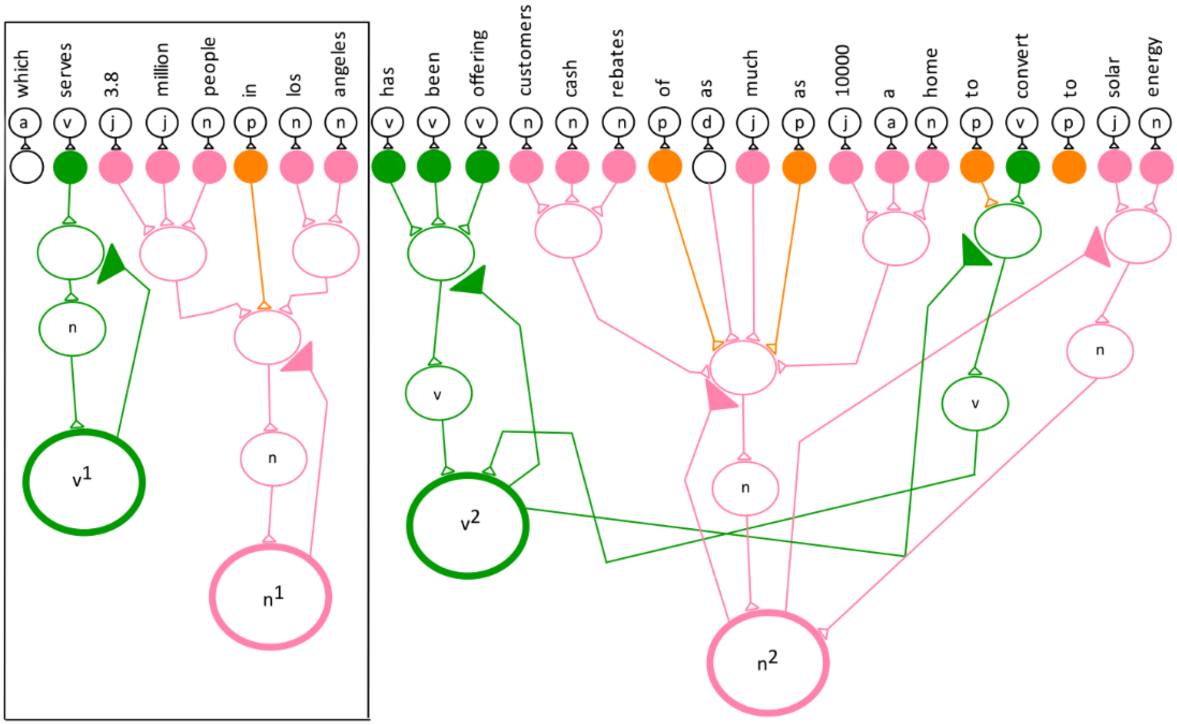

Figure 27. The divergence projection of the semantic neuronal network.

The Full Semantics

The full semantics of the sentence is:

Full semantics = $V^1 + N^1 + V^2 + N^2 + V^3 + N^3$

The verb and object patterns for this sentence is listed in Table 17.

General equation	The specific equation for each verb and noun phrase group
$V^1 + N^1$	Verb + Noun Phrase + Prepositional phrase
$V^2 + N^2$	Verb + Noun Phrase + Prepositional phrase
$V^3 + N^3$	Verb + Prepositional Phase

Table 17. The verb and its object for the full semantics.

The verb and noun phrase pattern for the sentence along with the words are listed in Table 18. Figure 28 depicts the entire sentence with its full semantics.

General equation	The specific equation for each verb and noun phrase group
$V^1 + N^1$	serves 3.8 million people in los angeles
$V^2 + N^2$	has been offering customers cash rebates of as much as 10000 a home
$V^3 + N^3$	to convert to solar energy.

Table 18. The actual phrases that make up the full semantic.

The full semantics for the sentence is:

the los angeles city utility, serves 3.8 million people in los angeles, has been offering customers cash rebates of as much as 10000 a home to convert to solar energy.

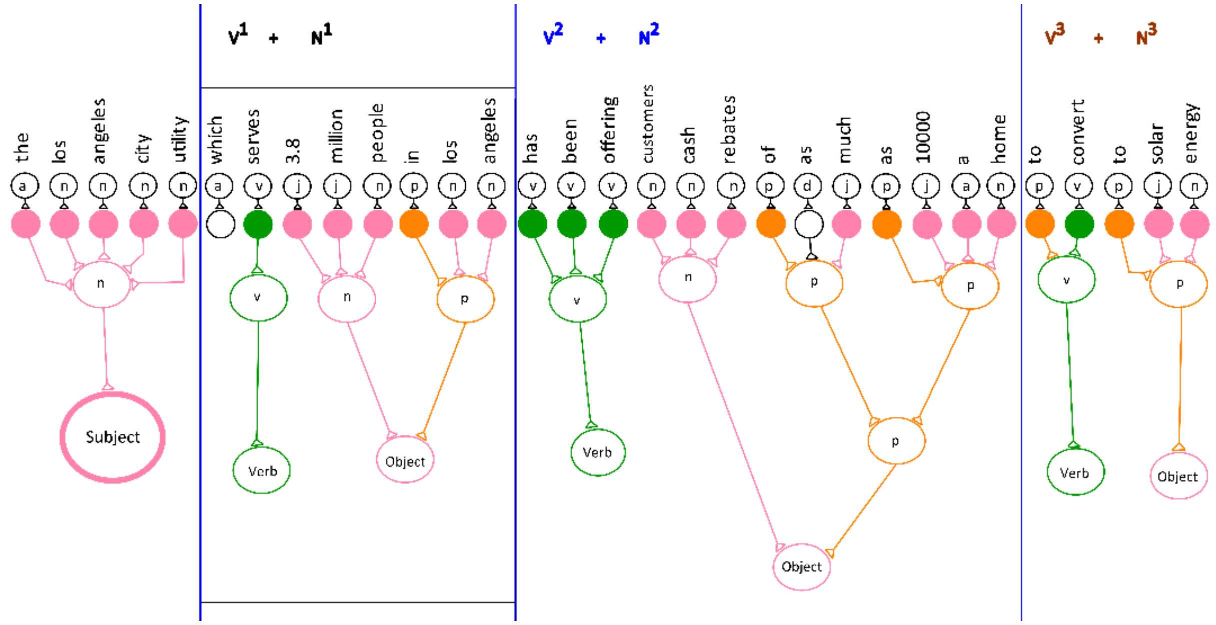

Figure 28. The full semantics for the fourth sentence.

The Partial Semantic I

The Partial Semantic I for the sentence is:

Partial Semantic I = $\text{Verb}^1 + noun_1^1 + noun_y^1 + \text{Verb}^2 + noun_1^2 + noun_y^2 + \text{Verb}^3 + noun_y^3$

Tables 19 and 19a show equations for each verb and noun phrase group within the sentence. Figure 29 shows the partial semantic I for the sentence.

Root Sentence	
**The specific equation**	**The corresponding words for the equation**
$\text{Verb}^2 + noun_1^2 + noun_y^2$	has been offering rebates homes
$\text{Verb}^3 + noun_y^3$	to convert to solar energy

Table 19. The phrases associated with the partial semantic I for the root sentence.

Subordinate Clause	
**The specific equation**	**The corresponding words for the equation**
$\text{Verb}^1 + noun_1^1 + noun_y^1$	serves people los angles

Table 19a. The phrases associated with the partial semantic I for the subordinate clause.

The partial semantic I for the sentence is:

the los angeles city utility serves people los angeles has been offering rebates home to convert solar energy.

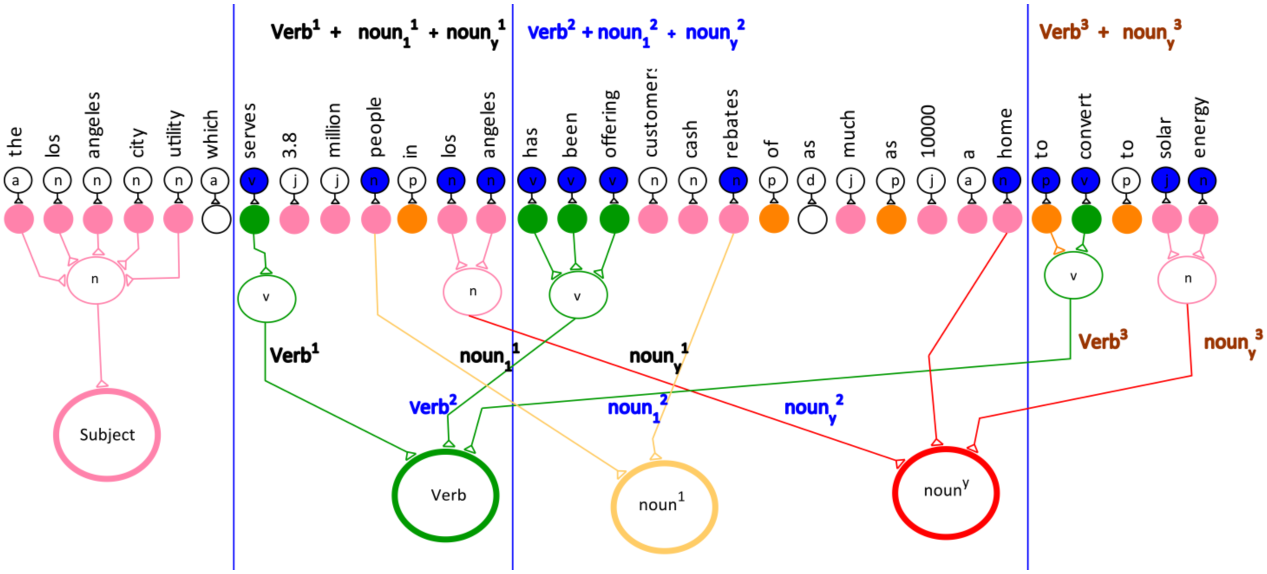

Figure 29. The partial semantic I for the fourth sentence.

The Partial Semantic III

Figure 30 shows the partial semantic III for the sentence.

Subject	Verb	Object/Complement
the los angeles city utility	has been offering	customers cash rebates of as much as 10000 a home

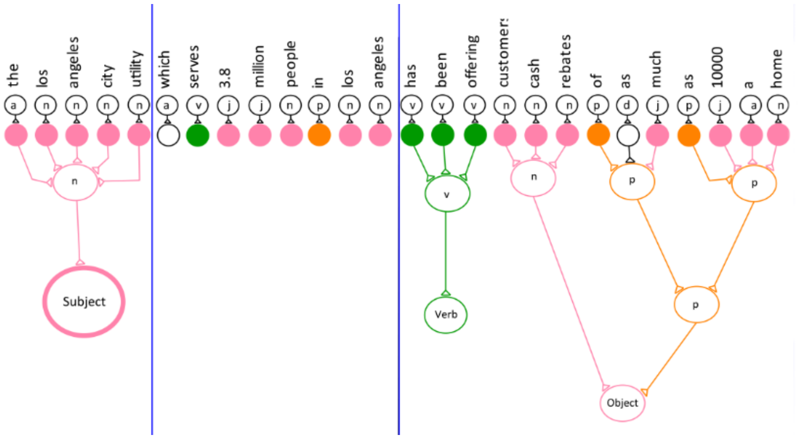

Figure 30. The partial semantic III for the fourth sentence.

EXAMPLE #5
Example #5 will demonstrate processing a long sentence. The sentence has fifty-nine words with three verb and noun phrase patterns.

Step 1: Determine the categories and identify the phrases in the sentence.

Most recently, physicists have been obsessed with trying to unify, or find connections among, the known fundamental forces of nature: gravity, electromagnetism, the "strong" force that holds particles together within the nucleus of an atom and the "weak" force that accounts for, among other things, radioactivity, the spontaneous disintegration of the nucleus that results in the emission of energy.

Each word in the sentence below is represented as a neuron.
physicists have been obsessed with trying to unify, or find connections among, the known fundamental forces of nature gravity, electromagnetism, the strong force that holds particles together within the nucleus of an atom and the weak force that accounts for, among other things, radioactivity, the spontaneous disintegration of the nucleus that results in the emission of energy.

Each category below is also represented as a neuron.
*noun verb verb verb prep verb prep verb * conjun verb noun prep * art adject adject noun prep noun noun * noun * art adject noun art verb noun adverb prep art noun prep art noun conjun art adject noun art verb prep * prep adject noun * noun * art adject noun prep art noun prep noun prep art noun prep noun*

Each category is represented as an extended ASCII character. The sentence is grouped into verb and noun phrases only in order to determine the number of each group.

Sentence = noun verb noun verb noun verb noun verb noun

The categories are grouped into an equation with plus signs as shown in Equation 17.

S = n + v + n + v + n + v + n

Equation 17. The equation for the entire semantic neuronal network.

Equation 17 and Figure 31 represent the entire semantic neuronal network with all its verb and noun phrases. Figure 31 represents the conceptual model of the entire sentence depicted as a semantic neuronal network.

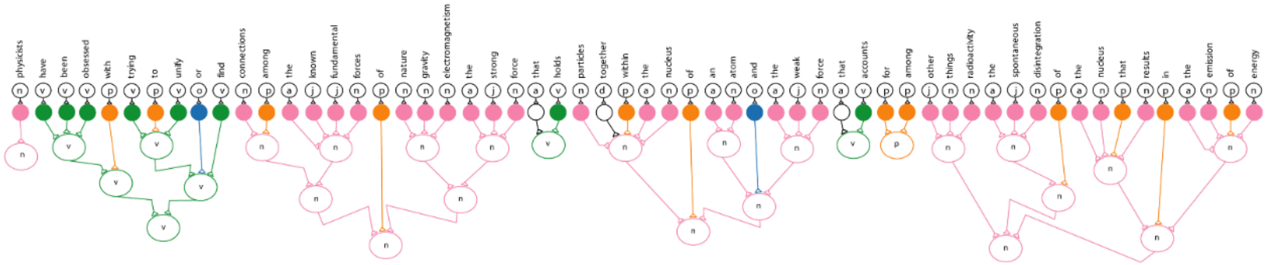

Figure 31. The entire semantic neuronal network.
Step 2: Locate the first verb, "have been obsessed" in the sentence.

Step 3: Determine the subject of the sentence and the words that may come before the first verb in the sentence.

Subject = physicists

Step 4: Remove the subject from the sentence.
Equation 18 and Figure 32 represent the semantic neuronal network after the subject was removed.

$$S = v + n + v + n + v + n$$

Equation 18. The equation for the semantic neuronal network without the subject.

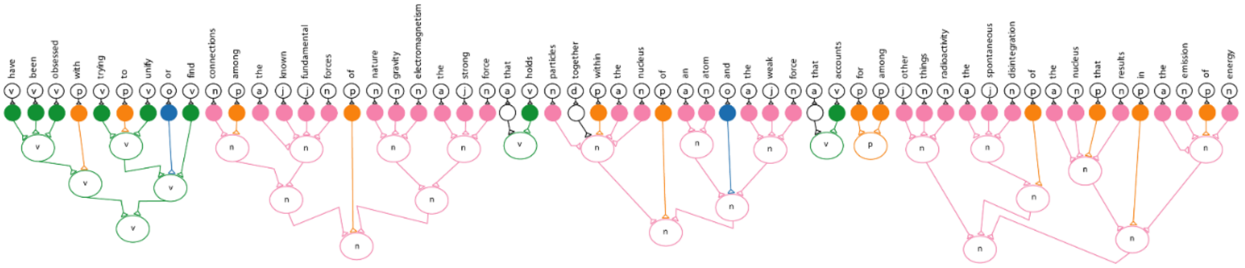

Figure 32. The semantic neuronal network for Equation 18.

The Frequency Technique

Step 5: Neurons summates using nonlinear summation.

The verb and noun phrases in Equation 18 are summed using nonlinear summation. Equation 19 and Figure 33 represent the semantic neuronal network as it depicts the frequency of the verb and noun phrases

$$S = v^3 + n^3$$

Equation 19. The frequency for both the verb and noun phrase.

Figure 33 represents the conceptual model of the convergence projection of the neuronal network that will be used to derive the frequency for both the verb and noun phrases.

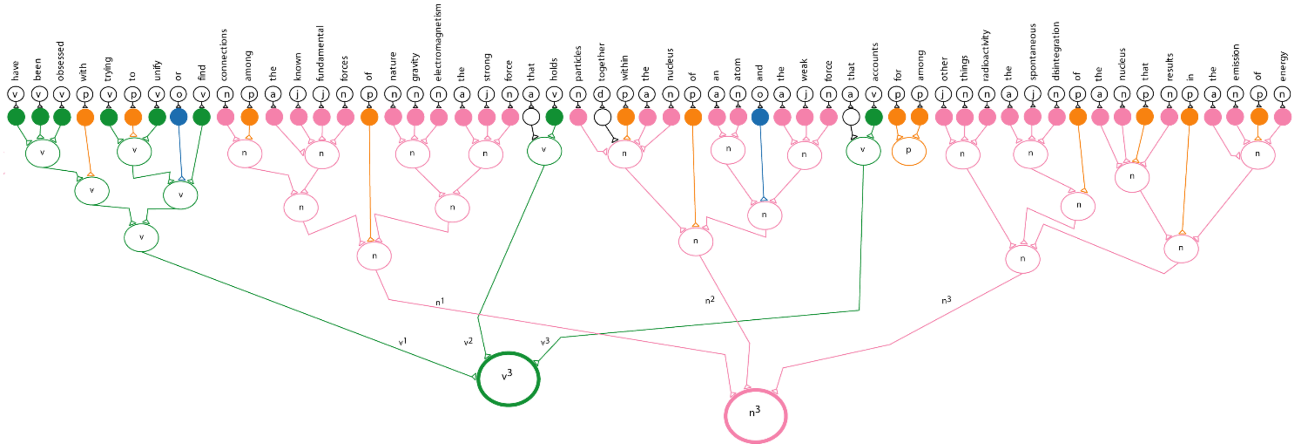

Figure 33. The convergence projection of the semantic neuronal network.

The Reduction Technique

Step 6: Reduce until the verb is in its lowest term (v^1).

Tables 20 shows the verb and noun phrase group for the sentence.
Figure 34 shows the reduction technique for the root sentence.

The reduction	The corresponding words for the verb and noun phrase
$S_0 = v^3 + n^3$	that accounts for, among other things, radioactivity, the spontaneous disintegration of the nucleus that results in the emission of energy.
$S_1 = v^2 + n^2$	that holds particles together within the nucleus of an atom and the weak force
$S_2 = v^1 + n^1$	have been obsessed with trying to unify, or find connections among, the known fundamental forces of nature gravity, electromagnetism, the strong force

Table 20. The verb and noun phrase patterns in the sentence.

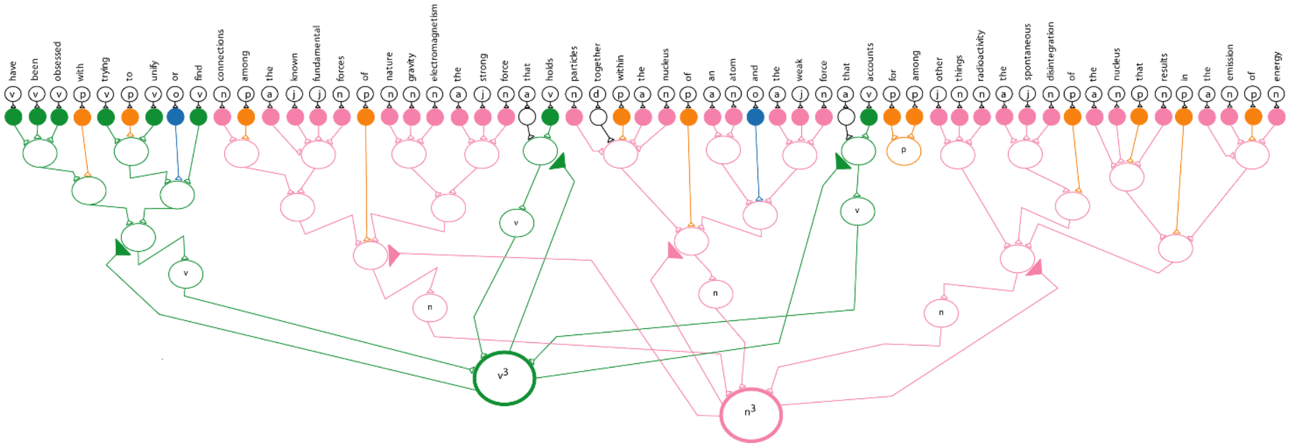

Figure 34. The divergence projection of the semantic neuronal network.

$$S = v^1 + n^1 + v^2 + n^2 + v^3 + n^3$$

Equation 20. The general equation.

The Full Semantics

The full semantics of the sentence is:

Full Semantics = $V^1 + N^1 + V^2 + N^2 + V^3 + N^3$

The verb and object patterns for this sentence is listed in Table 21.

General equation	The specific equation for each verb and noun phrase group
$V^1 + N^1$	Verb + Prepositional phrase
$V^2 + N^2$	Verb + Prepositional $Phase_1$ + Prepositional $Phase_3$ + Prepositional $Phase_5$
$V^3 + N^3$	Verb + Prepositional $Phase_1$

Table 21. The verb and its object for the full semantics.

The verb and noun phrase pattern for the sentence along with the words are listed in Table 22. Figure 35 depicts the entire sentence with its full semantics.

General equation	The corresponding words in the equation
$V^1 + N^1$	have been obsessed with trying to unify, or find connections among, the known fundamental forces
$V^2 + N^2$	that holds particles together within the nucleus
$V^3 + N^3$	that accounts for, among other things, radioactivity, the spontaneous disintegration that results of energy.

Table 22. The actual phrases that make up the full semantic.

The full semantics for the sentence is:

physicists have been obsessed with trying to unify, or find connections among, the known fundamental forces that holds particles together within the nucleus that accounts for, among other things, radioactivity, the spontaneous disintegration that results of energy

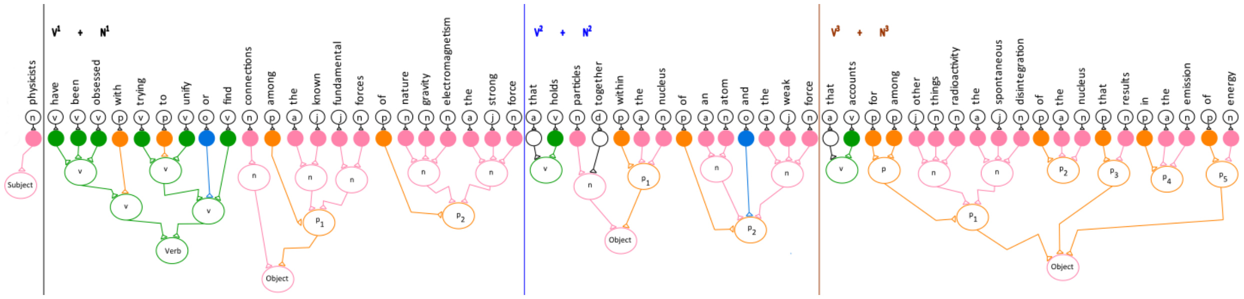

Figure 35. Full semantics for the fifth sentence.

The Partial Semantic I

The Partial Semantic I for the sentence is:

Partial Semantic I = $\text{Verb}^1 + noun_1^1 + noun_y^1 + \text{Verb}^2 + noun_1^2 + noun_y^2 + \text{Verb}^3 + noun_1^3 + noun_y^3$

Table 23 shows equations for each verb and noun phrase group within the sentence. Figure 36 shows the partial semantic I for the sentence.

The specific equation	The corresponding words for the equation
$\text{Verb}^1 + noun_1^1 + noun_y^1$	have been obsessed with trying to unify or find connections strong force
$\text{Verb}^2 + noun_1^2 + noun_y^2$	that holds particles together weak force
$\text{Verb}^3 + noun_1^3 + noun_y^3$	that accounts other things radioactivity emission of energy

Table 23. The phrases associated with the partial semantic I.

The partial semantic I for the sentence is:

physicists have been obsessed with trying to unify, or find connections strong force that holds particles together weak force that accounts other things radioactivity emission of energy

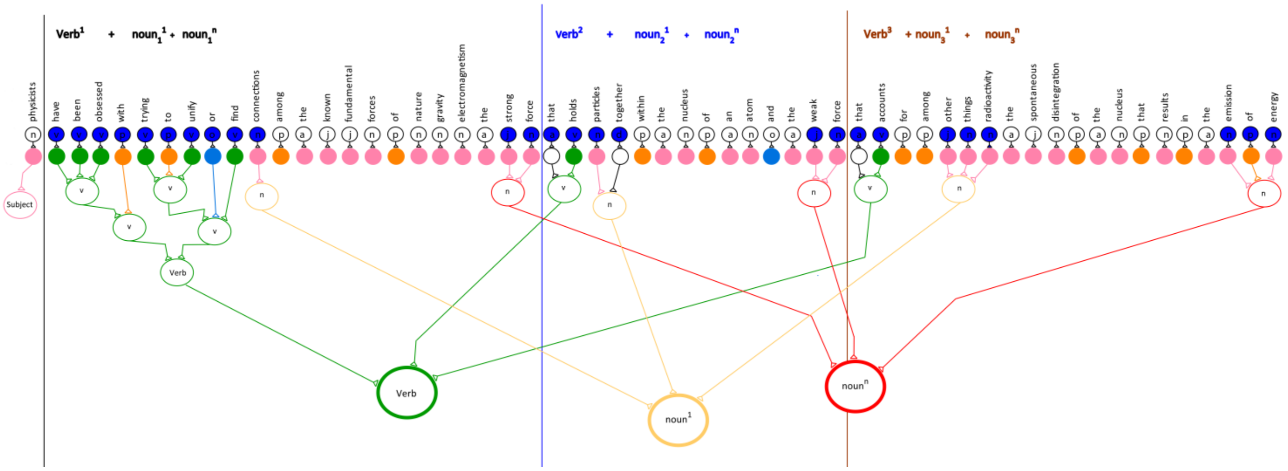

Figure 36. The partial semantics I for the fifth sentence.

The Partial Semantic III

Figure 37 shows the partial semantic III for the sentence.

Subject	Verb	Object
physicists	have been obsessed with trying to unify or find	connection among the known fundamental forces of nature

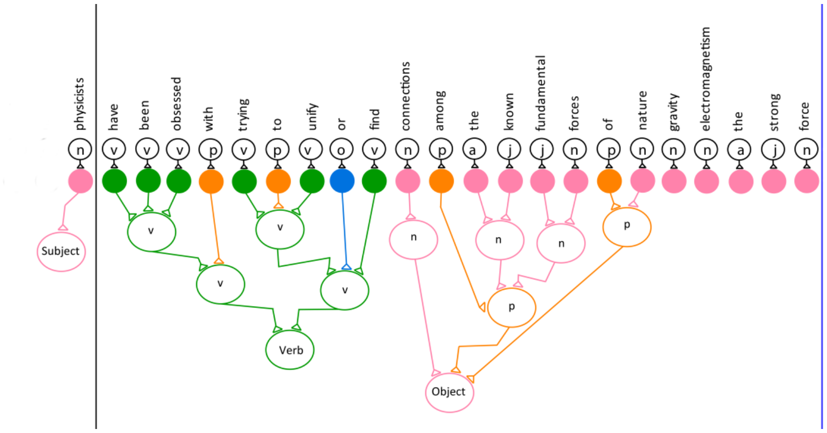

Figure 37. The partial semantic III for the fifth sentence.

For examples #6 and #7 the same sentence will used to demonstrate processing semantics with a subordinate clause and without a subordinate clause. The sentence has forty-nine words with four verb and noun phrase patterns.

EXAMPLE #6
Example #6 will demonstrate processing the sentence without the subordinate clause.

Step 1: Determine the categories and identify the phrases in the sentence.
Though unfinished, the history of health care legislation is a striking measure of the complexity of legislating major change in an era of intense partisanship, with a public that distrusts Washington as never before, a campaign technology applied to whipping around voters' opinions, and news reports that emphasize conflict, not explanation.

Each word in the sentence below is represented as a neuron.
unfinished, the history of health care legislation is a striking measure of the complexity of legislating major change in an era of intense partisanship, with a public that distrusts washington as never before, a campaign technology applied to whipping around voters, opinions, and news reports that emphasize conflict, not explanation.

Each category below is also represented as a neuron.
*adject * art noun prep noun noun noun verb art adject noun prep art noun prep noun adject noun prep art noun prep adject noun * prep art noun art verb noun adverb adverb adverb * art noun noun verb prep adject prep noun * noun * conjun noun noun art verb noun * adverb noun*

Each category is represented as an extended ASCII character. The sentence is grouped into verb and noun phrases only in order to determine the number of each group.

Sentence = noun verb noun verb noun verb noun verb noun
The categories are grouped into an equation with plus signs as shown in Equation 21.

S = n + v + n + v + n + v + n + v + n

Equation 21. The equation for the entire semantic neuronal network.

Equation 21 and Figure 38 represent the entire semantic neuronal network with all its verb and noun phrases. Figure 38 represents the conceptual model of the entire sentence depicted as a semantic neuronal network.

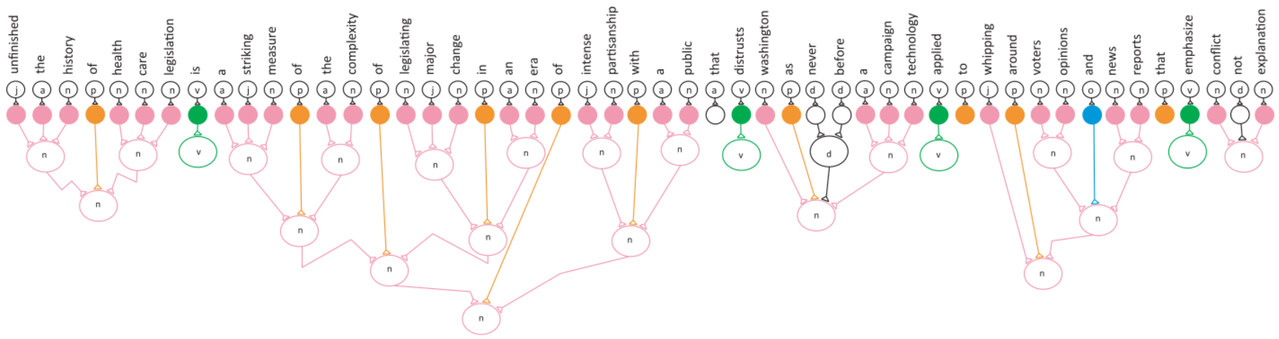

Figure 38. The entire semantic neuronal network.

Step 2: Locate the first verb, “is” in the sentence.

Step 3: Determine the subject of the sentence and the words that may come before the first verb in the sentence.

Subject = unfinished the history of health care legislation

Step 4: Remove the subject from the sentence.

Equation 22 and Figure 39 represent the semantic neuronal network after the subject was removed.

$$S = v + n + v + n + v + n + v + n$$

Equation 22. The equation for the semantic neuronal network without the subject.

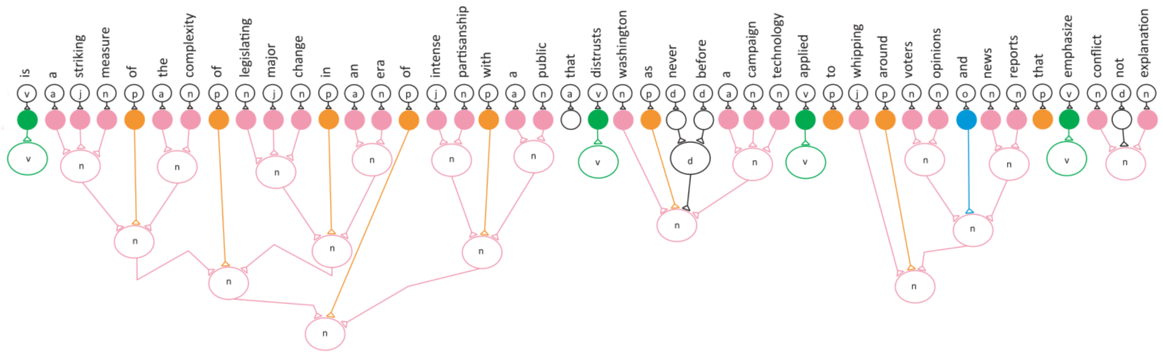

Figure 39. The semantic neuronal network for Equation 26.

The Frequency Technique

Step 5: Neurons summates using nonlinear summation.

The verb and noun phrases in Equation 22 are summed using nonlinear summation.

Equation 23 and Figure 40 represent the semantic neuronal network as it depicts the frequency of the verb and noun phrases.

$$S_0 = v^4 + n^4$$

Equation 23. The frequency for both the verb and noun phrase.

Figure 40 represents the conceptual model of the convergence projection of the neuronal network that will be used to derive the frequency for both the verb and noun phrases.

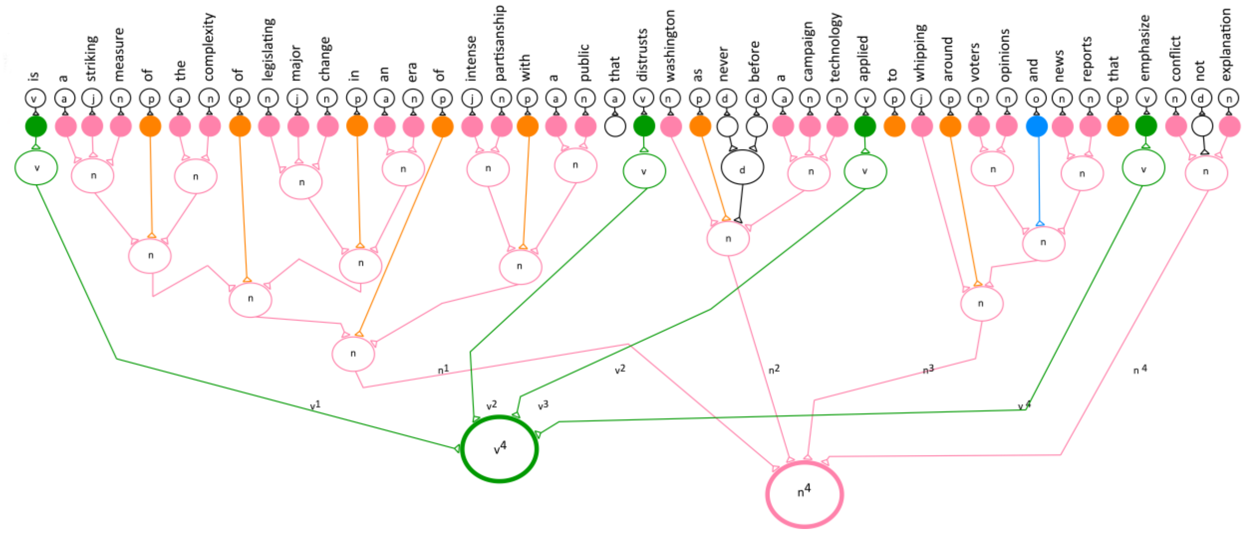

Figure 40. The convergence projection of the semantic neuronal network.

The Reduction Technique

Step 6: Reduce until the verb is in its lowest term (v^1).

Table 24 shows the verb and noun phrase group for the sentence, where $\mathbf{S = v^4 + n^4}$ points to the last verb and noun phrase group in the sentence. Figure 41 depicts the divergence projection of the neuronal network for the entire sentence.

The reduction	The corresponding words for the verb and noun phrase
$S_0 = v^4 + n^4$	that emphasize conflict, not explanation
$S_1 = v^3 + n^3$	applied to whipping around voters, opinions, and news reports
$S_2 = v^2 + n^2$	that distrusts washington as never before, a campaign technology
$S_3 = v^1 + n^1$	is a striking measure of the complexity of legislating major change in an era of intense partisanship, with a public

Table 24. The verb and noun phrase patterns in the sentence.

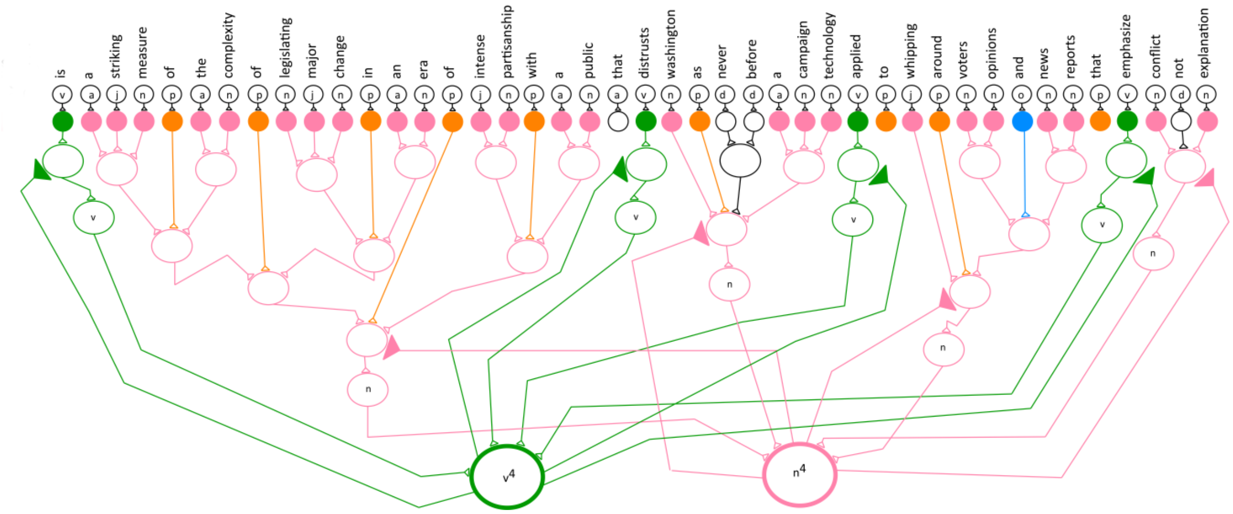

Figure 41. The divergence projection of the semantic neuronal network.

$$S = v^1 + n^1 + v^2 + n^2 + v^3 + n^3 + v^4 + n^4$$

Equation 24. The general equation.

The Full Semantics

The full semantics of the sentence is:

Full Semantics = $V^1 + N^1 + V^2 + N^2 + V^3 + N^3 + V^4 + N^4$

The verb and object patterns for this sentence is listed in Table 25.

General equation	The specific equation for each verb and noun phrase group
$V^1 + N^1$	Verb + Noun Phrase + Prepositional Phrase$_1$ + Prepositional Phrase$_3$ + Prepositional Phrase$_3$
$V^2 + N^2$	Verb + Noun + Prepositional Phrase
$V^3 + N^3$	Verb + Prepositional Phrase + Prepositional Phrase + Preposition
$V^4 + N^4$	Verb + Noun Phrase

Table 25. The verb and its object for the full semantics.

Figure 42 depicts the entire sentence with its full semantics.

The verb and noun group patterns along with the corresponding words for the sentence are listed in Table 26.

General equation	The corresponding words in the equation
$V^1 + N^1$	is a striking measure of the complexity of legislating major change in an era of intense partisanship, with a public
$V^2 + N^2$	that distrusts washington as never before, a campaign technology
$V^3 + N^3$	applied to whipping around voters, opinions, and news reports
$V^4 + N^4$	emphasize conflict, not explanation

Table 26. The actual phrases that make up the full semantic.

The full semantics for the sentence is:

unfinished the history of health care legislation is a striking measure of the complexity in an era with a public that distrusts washington as never before, a campaign technology applied to whipping around voters, opinions, and news reports emphasize conflict, not explanation.

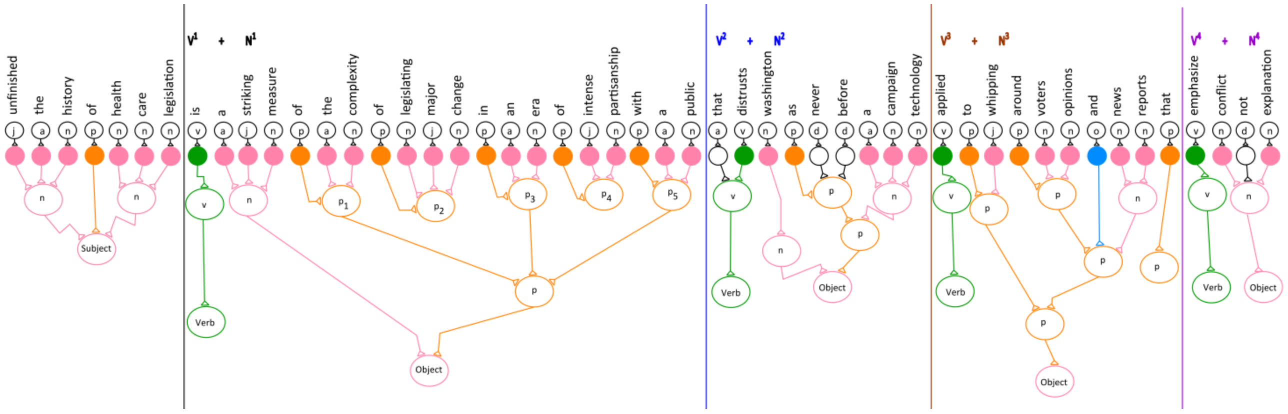

Figure 42. The full semantics for the seventh sentence.

The Partial Semantic I

The Partial Semantic I for the sentence is:

Partial Semantic I = $\text{Verb}^1 + noun_1^1 + noun_y^1 + \text{Verb}^2 + noun_1^2 + noun_y^2 + \text{Verb}^3 + noun_1^3 + noun_y^3 +$
$\text{Verb}^4 + noun_1^4 + noun_y^4$

Table 27 shows the equations for each verb and noun phrase group within the sentence. Figure 43 shows the partial semantic I for the sentence.

The specific equation	The corresponding words for the equation
$\text{Verb}^1 + noun_1^1 + noun_y^1$	is a striking measure a public
$\text{Verb}^2 + noun_1^2 + noun_y^2$	that distrust washington a campaign
$\text{Verb}^3 + noun_1^3 + noun_y^3$	applied voters opinions news reports
$\text{Verb}^4 + noun_1^4 + noun_y^4$	emphasize conflict not explanation

Table 27. The phrases associated with the partial semantic I.

The partial semantic I for the sentence:

unfinished the history of health care legislation is a striking measure a public that distrusts washington a campaign technology applied voters opinions news reports emphasize conflict not explanation.

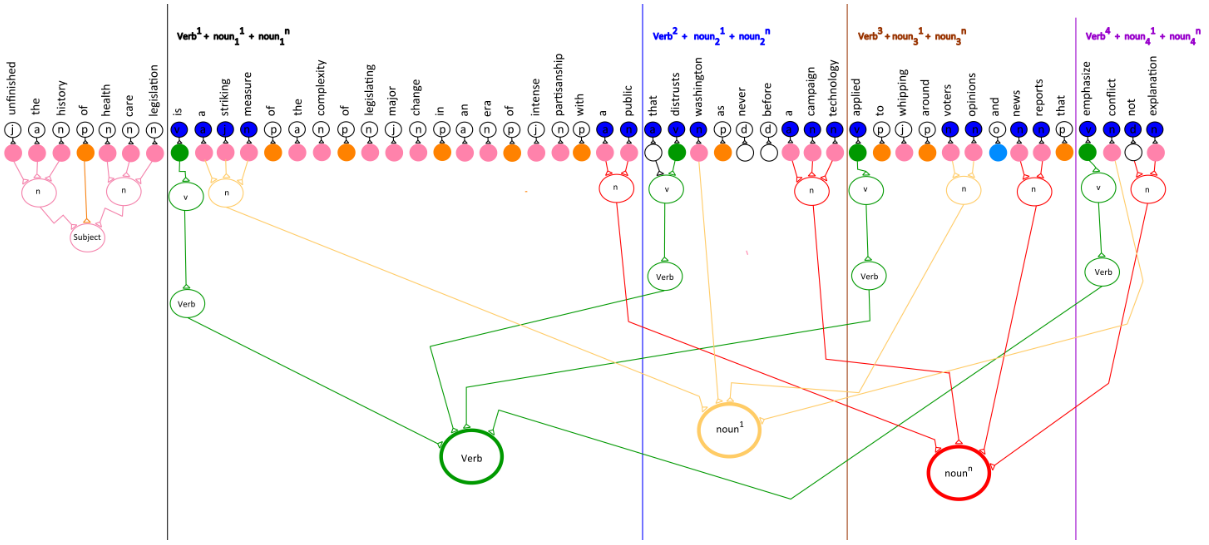

Figure 43. The partial semantic I for the seventh sentence.

The Partial Semantic III

Figure 44 shows the partial semantic III for the sentence.

Subject	Verb	Object/Complement
unfinished the history of health care legislation	is	a striking measure of the complexity in an era of with a public

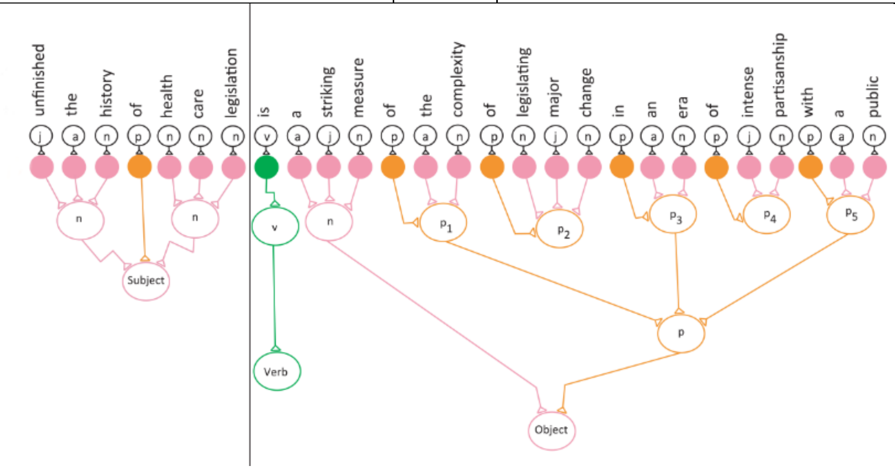

Figure 44. The partial semantic III for the seventh sentence.

Figure 45 shows the partial semantic III for the sentence.

Subject	Verb	Object/Complement
unfinished the history of health care legislation	is	a striking measure of legislating major change in an era of intense partisanship

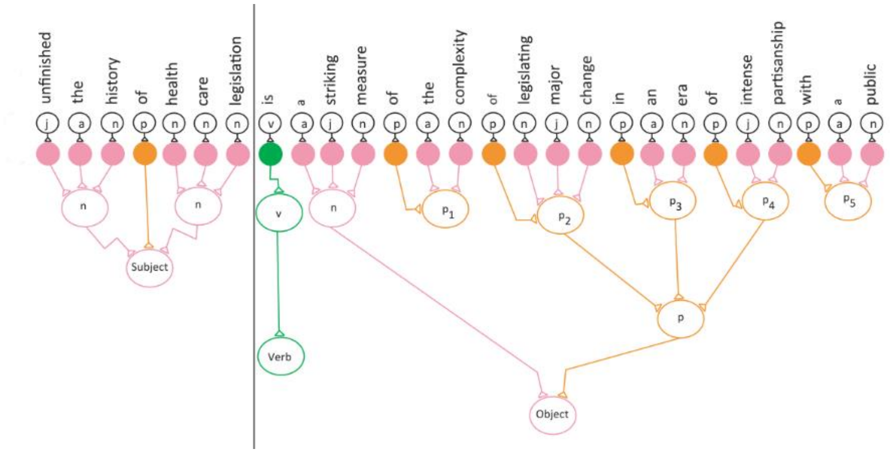

Figure 45. The partial semantic III for the seventh sentence.

Figure 46 shows the partial semantic III for the sentence.

Subject	Verb	Object/Complement
unfinished the history of health care legislation	is	a striking measure of the complexity in an era of intense partisanship

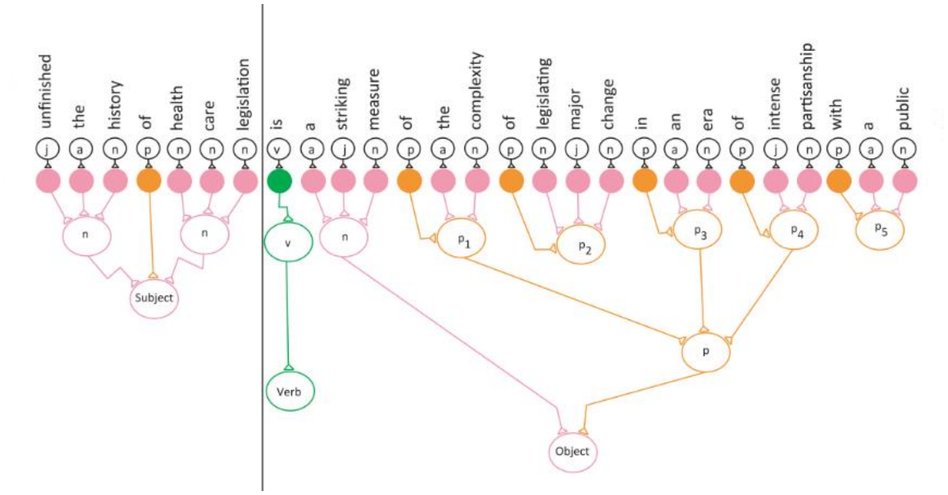

Figure 46. Partial semantics III for the seventh sentence.

EXAMPLE #7

Example #7 will demonstrate processing with a subordinate clause. The sentence has forty-nine words with four verb and noun phrase patterns.

Step 1: Determine the categories and identify the phrases in the sentence.

Though unfinished, the history of health care legislation is a striking measure of the complexity of legislating major change in an era of intense partisanship, with a public that distrusts Washington as never before, a campaign technology applied to whipping around voters' opinions, and news reports that emphasize conflict, not explanation.

Each word in the sentence below is represented as a neuron.

unfinished, the history of health care legislation is a striking measure of the complexity of legislating major change in an era of intense partisanship, with a public that distrusts washington as never before, a campaign technology applied to whipping around voters, opinions, and news reports that emphasize conflict, not explanation.

Each category below is also represented as a neuron.

*adject * art noun prep noun noun noun verb art adject noun prep art noun prep noun adject noun prep art noun prep adject noun * prep art noun art verb noun adverb adverb adverb * art noun noun verb prep adject prep noun * noun * conjun noun noun art verb noun * adverb noun*

Each category is represented as an extended ASCII character. The sentence is grouped into verb and noun phrases only in order to determine the number of each group.

Sentence = noun verb noun verb noun verb noun verb noun
The categories are grouped into an equation with plus signs as shown in Equation 25.

$$S = n + v + n + v + n + v + n$$

Equation 25. The equation for the entire semantic neuronal network.

Equation 25 and Figure 47 represent the entire semantic neuronal network with all its verb and noun phrases. Figure 47 represents the conceptual model of the entire sentence depicted as a semantic neuronal network.

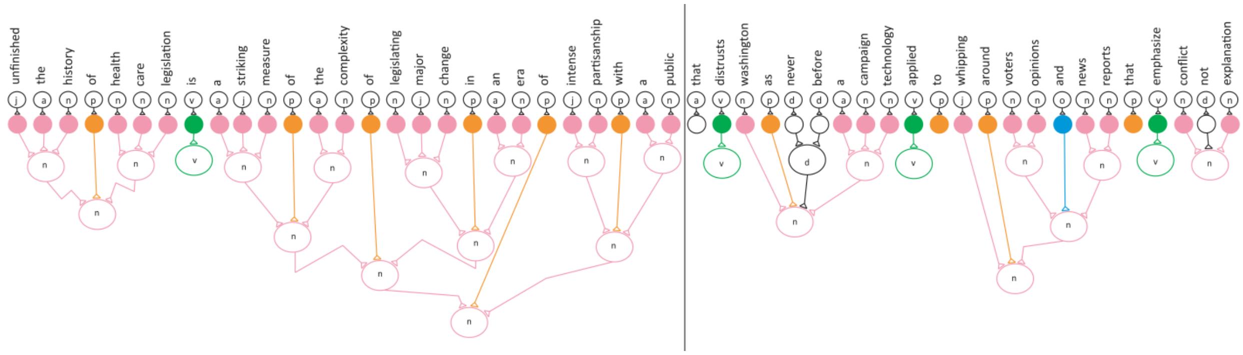

Figure 47. The entire semantic neuronal network.

Step 2: Locate the first verb, “is” in the sentence.

Step 3: Determine the subject of the sentence and the words that may come before the first verb in the sentence.

Subject = unfinished the history of health care legislation

Step 4: Remove the subject from the sentence.

Equation 26 and Figure 48 represent the semantic neuronal network after the subject was removed.

$$S = v + n + v + n + v + n$$

Equation 26. The equation for the semantic neuronal network without the subject.

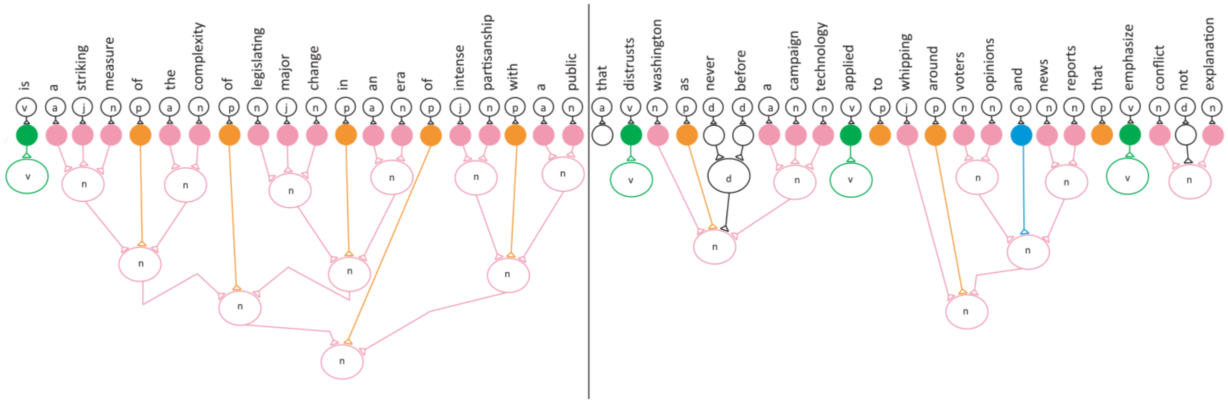

Figure 48. Semantic neuronal network for Equation 55.

The Frequency Technique

Step 5: Neurons summates using nonlinear summation.

The verb and noun phrases in Equation 26 are summed using nonlinear summation. Equation 27 and Figure 49 represent the semantic neuronal network as it depicts the frequency of the verb and noun phrases.

$S_0 = v^3 + n^3$

Equation 27. The frequency for both the verb and noun phrase.

Figure 49 represents the conceptual model of the convergence projection of the neuronal network that will be used to derive the frequency for both the verb and noun phrases.

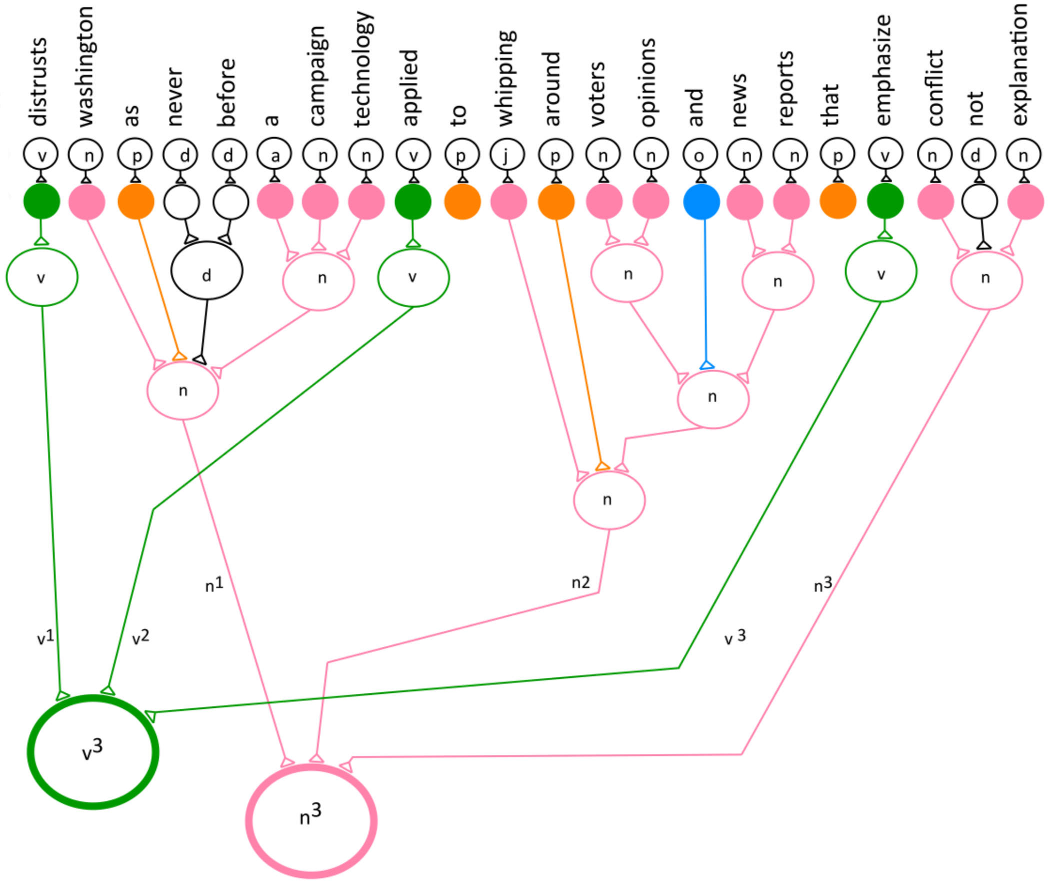

Figure 49. The convergence projection of the semantic neuronal network.

The Reduction Technique

Step 6: Reduce until the verb is in its lowest term (v1).

Table 28 and 28a show the verb and noun phrase group for the sentence.
Figure 50 shows the reduction technique for subordinate clause.

Root Sentence	
**The reduction**	**The corresponding words for the verb and noun phrase**
$S_0 = v^1 + n^1$	is a striking measure of the complexity of legislating major change in an era of intense partisanship, with a public

Table 28. The reduction techninque for the root sentence.

Subordinate Clause	
**The reduction**	**The corresponding words for the verb and noun phrase**
$S_0 = v^3 + n^3$	emphasize conflict, not explanation
$S_1 = v^2 + n^2$	applied to whipping around voters, opinions, and news reports that
$S_2 = v^1 + n^1$	that distrusts washington as never before, a campaign technology

Table 28a. The reduction techninque for the subordinate clause.

$$S_0 = v^1 + n^1$$

Equation 28. The general equation.

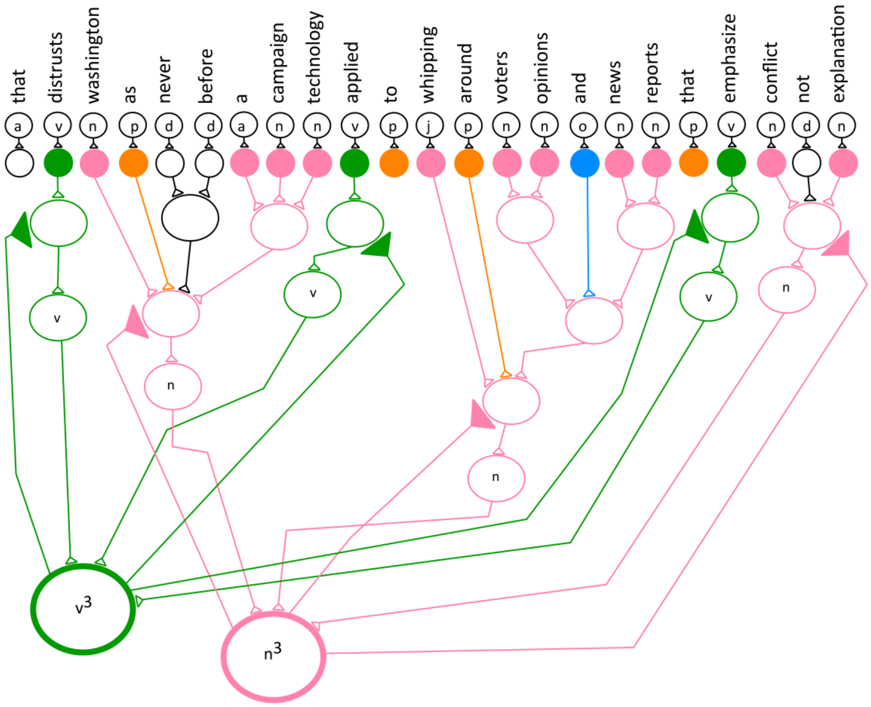

Figure 50. The divergence projection of the semantic neuronal network.

$$S = v^1 + n^1 + v^2 + n^2 + v^3 + n^3$$

Equation 28a. The general equation.

The Full Semantics

The full semantics of the sentence is:

Full Semantics = $V^1 + N^1 + V^2 + N^2 + V^3 + N^3 + V^4 + N^4$

The verb and object patterns for this sentence is listed in Table 29.

Root Sentence	
**General equation**	**The specific equation for each verb and noun phrase group**
$V^1 + N^1$	Verb + Noun Phrase + Prepositional Phrase$_1$ + Prepositional Phrase$_3$ + Prepositional Phrase$_3$

Table 29. The verb and its object for the full semantics.

Subordinate Clause	
**General equation**	**The specific equation for each verb and noun phrase group**
$V^1 + N^1$	Verb + Noun Phrase + Prepositional Phase
$V^2 + N^2$	Verb + Prepositional Phase + Prepositional Phase
$V^3 + N^3$	Verb + Noun Phrase

Table 29a. The verb and its object for the full semantics.

The verb and object patterns for this sentence is listed in Tables 30 and 30a.

Root Sentence	
**General equation**	**The corresponding words in the equation**
$V^1 + N^1$	is a striking measure of the complexity in an era with a public

Table 30. The actual phrases that make up the full semantic for the root sentence.

Figure 51 shows the full semantic for the sentence.

Subordinate Clause	
**General equation**	**The corresponding words in the equation**
$V^1 + N^1$	that distrusts washington as never before, a campaign technology
$V^2 + N^2$	applied to whipping around voters, opinions, and news reports
$V^3 + N^3$	emphasize conflict, not explanation

Table 30a. The actual phrases that make up the full semantic for the subordinate clause.

The full semantics for the sentence is:

unfinished the history of health care legislation is a striking measure of the complexity in an era with a public that distrusts washington as never before, a campaign technology applied to whipping around voters, opinions, and news reports emphasize conflict, not explanation.

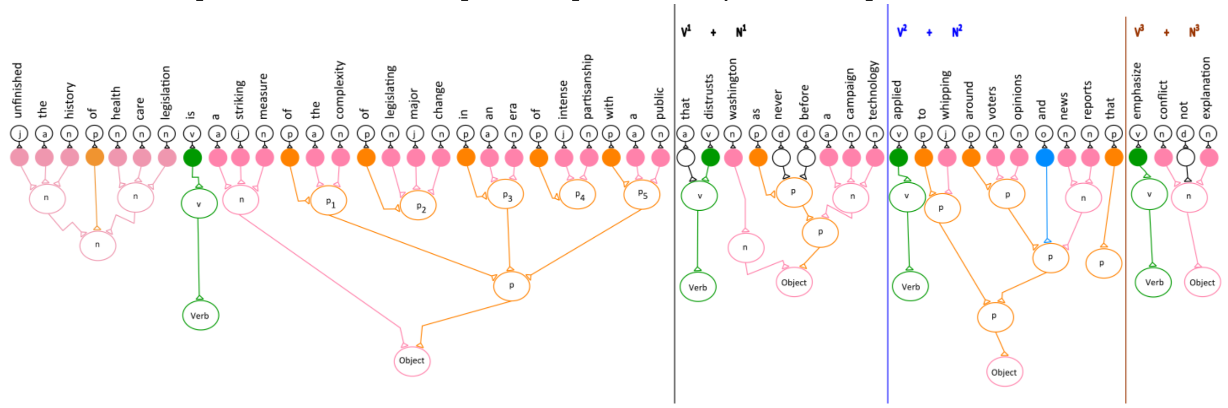

Figure 51. The full semantics for the eighth sentence.

The Partial Semantic I

The Partial Semantic I for the sentence is:

$$\text{Partial Semantic I} = \text{Verb}^1 + noun_1^1 + noun_y^1 + \text{Verb}^2 + noun_1^2 + noun_y^2 + \text{Verb}^3 + noun_1^3 + noun_y^3 + \text{Verb}^4 + noun_1^4 + noun_y^4$$

Tables 31 and 31a show the equations for each verb and noun phrase group within the sentence. Figure 52 shows the partial semantic I for the sentence.

Root Sentence	
**The specific equation**	**The corresponding words for the equation**
$Verb^1 + noun_1^1 + noun_y^1$	is a striking measure a public

Table 31. The phrases associated with the partial semantic I for the root sentence.

Subordinate Clause	
**The specific equation**	**The corresponding words for the equation**
$Verb^2 + noun_1^2 + noun_y^2$	that distrust washington a campaign
$Verb^3 + noun_1^3 + noun_y^3$	applied voters opinions news reports
$Verb^4 + noun_1^4 + noun_y^4$	emphasize conflict not explanation

Table 31a. The phrases associated with the partial semantic I for the subordinate clause.

The partial semantic I for the sentence is:

unfinished the history of health care legislation is a striking measure a public that distrusts washington a campaign technology applied voters opinions news reports emphasize conflict not explanation.

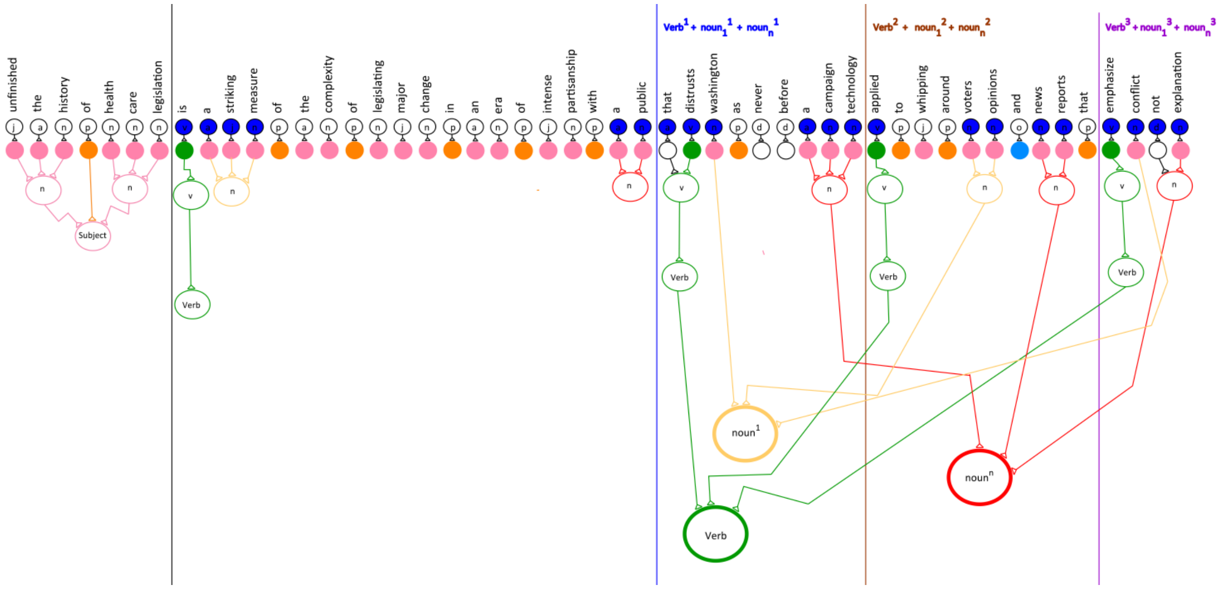

Figure 52. The partial semantic I for the eighth sentence.

The Partial Semantic III

Figure 53 shows the partial semantic III for the sentence.

Subject	Verb	Object/Complement
unfinished the history of health care legislation	is	is a striking measure of the complexity in an era with a public
	that distrusts	washington

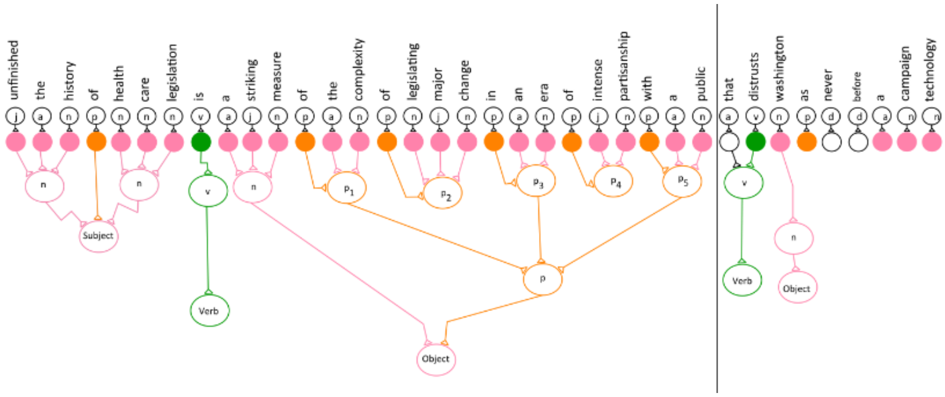

Figure 53. The partial semantics III for the eighth sentence.
